# Prediction, Postdiction, and Perceptual Length Contraction: A Bayesian Low-Speed Prior Captures the Cutaneous Rabbit and Related Illusions

**DOI:** 10.3389/fpsyg.2013.00221

**Published:** 2013-05-10

**Authors:** Daniel Goldreich, Jonathan Tong

**Affiliations:** ^1^Department of Psychology, Neuroscience & Behaviour, McMaster UniversityHamilton, ON, Canada

**Keywords:** probabilistic inference, sensory saltation, motion illusions, tactile spatial attention, optimal percepts, Kalman smoothing, somatosensory spatiotemporal perception, sensory uncertainty

## Abstract

Illusions provide a window into the brain’s perceptual strategies. In certain illusions, an ostensibly task-irrelevant variable influences perception. For example, in touch as in audition and vision, the perceived distance between successive punctate stimuli reflects not only the actual distance but curiously the inter-stimulus time. Stimuli presented at different positions in rapid succession are drawn perceptually toward one another. This effect manifests in several illusions, among them the startling cutaneous rabbit, in which taps delivered to as few as two skin positions appear to hop progressively from one position to the next, landing in the process on intervening areas that were never stimulated. Here we provide an accessible step-by-step exposition of a Bayesian perceptual model that replicates the rabbit and related illusions. The Bayesian observer optimally joins uncertain estimates of spatial location with the expectation that stimuli tend to move slowly. We speculate that this expectation – a Bayesian prior – represents the statistics of naturally occurring stimuli, learned by humans through sensory experience. In its simplest form, the model contains a single free parameter, tau: a time constant for space perception. We show that the Bayesian observer incorporates both pre- and post-dictive inference. Directed spatial attention affects the prediction-postdiction balance, shifting the model’s percept toward the attended location, as observed experimentally in humans. Applying the model to the perception of multi-tap sequences, we show that the low-speed prior fits perception better than an alternative, low-acceleration prior. We discuss the applicability of our model to related tactile, visual, and auditory illusions. To facilitate future model-driven experimental studies, we present a convenient freeware computer program that implements the Bayesian observer; we invite investigators to use this program to create their own testable predictions.

## Introduction

**Figure 1:**
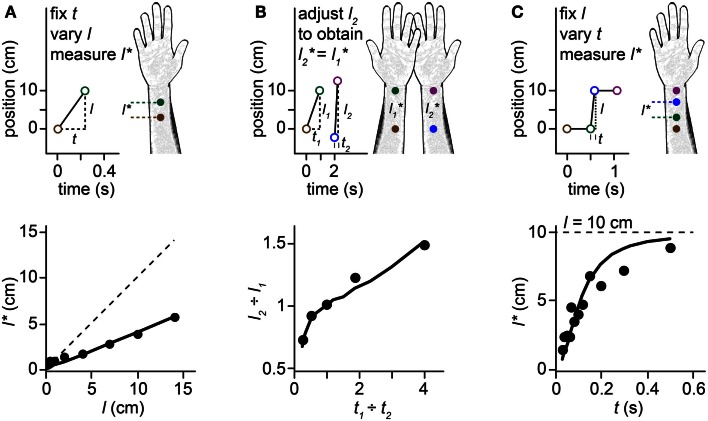
**Perceptual length contraction**. Perception underestimates the distance between successive taps to the skin. Stimuli on the forearm are illustrated in the upper panels, along with their perception (forearm sketches). Corresponding human data and Bayesian model fits are plotted in the lower panels. In this and subsequent figures, we illustrate stimulus sequences that progress distally on the arm; the illusions occur also for stimuli in the opposite direction. **(A)** *Top*: at short ISI (*t*), the perceived length (l*) between two taps to the forearm is less than the actual length (*l*). *Bottom*: perceived length grows linearly with actual length, but with a slope less than 1. Filled circles: human perceptual data from Marks et al. (1982) for electrocutaneous stimuli delivered at *t* = 0.24 s. Solid line: fit of the Bayesian model. Dashed line: *l* = l*. **(B)** *Top*: a pair of taps delivered to the right forearm at short ISI (*t*_2_) is perceived to have the same spacing as a more closely spaced pair of taps (*l*_1_ < *l*_2_) delivered to the left forearm at longer ISI (*t*_1_ > *t*_2_). *Bottom*: the spacing ratio, *l*_2_-to-*l*_1_, resulting in perceived equality of spacing on the two arms, as a function of the ISI ratio, *t*_1_-to-*t*_2_. Filled circles: human perceptual data from Lechelt and Borchert (1977). Curve: fit of the Bayesian model. Data points from left to right had *t*_1_ = 0.2, 0.35, 0.5, 0.65, and 0.8 s, with *t*_2_ = 1.0 s − *t*_1_, and *l*_1_ = 10 cm. **(C)** *Top*: 4 taps delivered to two skin sites are perceived as hopping sequentially along the arm, because the short ISI (*t*) between taps 2 and 3 results in contraction of the perceived distance between them (l* < *l*). *Bottom*: the perceived length from taps 2–3 asymptotically approaches the actual length (*l* = 10 cm, dashed line) as ISI is increased. Filled circles: human perceptual data from Kilgard and Merzenich (1995). Curve: fit of the Bayesian model.

Illusions provide investigators a window into the brain’s unconscious perceptual strategies. In a particularly interesting category of illusions, an ostensibly task-irrelevant stimulus feature strongly influences the perception of a target feature. Here we consider one group of such illusions, characterized by the curious influence of time on the tactile perception of space (Figure 1).

When humans are asked to judge the distance between two brief taps delivered in rapid succession to the skin, they consistently underestimate the true distance. Indeed, the perceived distance between taps shortens systematically as the time between taps is reduced. This *perceptual length contraction* occurs even when the participant is explicitly instructed to attend only to the distance between stimuli, and to ignore the time. The phenomenon is particularly pronounced on the forearm and other body areas that have poor spatial acuity. Several striking illusions result from this puzzling compressive effect of time on space perception (Figures 1A–C). For instance, a stimulus sequence consisting of two-taps delivered at one position followed by two taps at another, with a short inter-stimulus interval (ISI) separating the second and third taps, is perceived as four taps hopping progressively along the arm: the second and third taps are perceptually displaced from their true positions, as if attracted toward one another (Figure 1C). This phenomenon is known as sensory saltation, or more famously, the cutaneous rabbit illusion (Geldard and Sherrick, 1972; Geldard, 1982). Analogous phenomena occur in vision (Geldard, 1976; Lockhead et al., 1980; Khuu et al., 2011) and audition (Bremer et al., 1977; Shore et al., 1998; Getzmann, 2009).

Why does time influence space perception in this manner? Much research supports the view that perception works out a probabilistic best guess. An optimal probabilistic (i.e., Bayesian) observer interprets the current sensory input, not in isolation, but rather within the context of the structure and statistics of the natural world (Knill and Pouget, 2004; Vilares and Kording, 2011). By exploiting its knowledge of the world, the observer achieves a more accurate perceptual inference. Following the Bayesian model of Goldreich (2007), we hypothesize that perception interprets successive taps to the skin as arising from a moving object that touches down intermittently, and that perception expects slowly moving objects to occur more often than rapidly moving ones. We speculate that the expectation for slow movement results from a lifetime of experience with tactile stimuli that are primarily stationary (e.g., the pressure of clothing against the skin) or – somewhat less frequently – slowly moving (e.g., grooming, movement of clothing during walking, etc.). Thus, in the observer’s experience, stimuli separated by large distances at short ISI are uncommon. Faced with such a stimulus sequence, and somewhat uncertain as to the true locations of the taps, the brain concludes that the sensory measurements were caused by a stimulus sequence that was more probable *a priori*: one that moved at a slower speed (i.e., shorter distance) on the skin. Under this view, the influence of time over space perception, far from reflecting a design flaw in our perceptual machinery, is a consequence of optimal probabilistic inference under conditions of sensory uncertainty.

Here, we present and elaborate on the Bayesian observer model introduced by Goldreich (2007). We show that our model is compatible with the view that the rabbit illusion – and perceptual length contraction generally – involves concomitant pre- and postdiction. By prediction, we mean an inference process in which earlier sensory events influence the perception of later ones. By postdiction, we mean an inference process in which later sensory events influence the perception of earlier ones (Eagleman and Sejnowski, 2000). We show interestingly that pre- and postdiction emerge naturally from our model, even though the model does not explicitly represent these processes. We show further that directed spatial attention shifts the Bayesian observer’s percept by modulating the prediction-postdiction balance. Finally, we apply our Bayesian model to the perception of spatiotemporal stimulus patterns that are more complex than those depicted in Figure 1.

## The Fundamentals of the Bayesian Observer

Stochastic variability in stimulus-evoked neural activity presents one of many challenges to perception. An identical repeated stimulus – such as a tap to a particular location on the skin – will evoke a different neural response on each trial (Sripati et al., 2006). Consequently, a given response could have been caused by a stimulus at any one of many locations. The spatial uncertainty caused by stochastic variability is lessened, but not eliminated, when a stimulus activates a larger number of neurons. On the forearm, where receptor density is relatively low, humans can localize a stimulus to within about ±1 cm of its true location; on the fingertip, where receptor density is much higher, localization improves to about ±1 mm (Weinstein, 1968).

To model stochastic neural variability, we assume that a single tap to the skin evokes an internal position *measurement* that is randomly sampled from a Gaussian distribution centered at the true tap position, with a standard deviation, σ*_s_*, that depends on the receptor density (the subscript *s* signifies “spatial”)^1^. On repeated trials with an identical tap position, the measurement will vary stochastically, but on average will equal the true position. In the absence of any other perceptual influence, the measurement is the location the observer perceives. Consequently, on average the perception of an isolated single tap to the skin is veridical. However, unlike an isolated single tap, a rapid spatiotemporal tap sequence is not veridically perceived (Figure 1). To understand why, we explore a probabilistic model – a Bayesian observer that makes a perceptual best guess.

**Figure 2:**
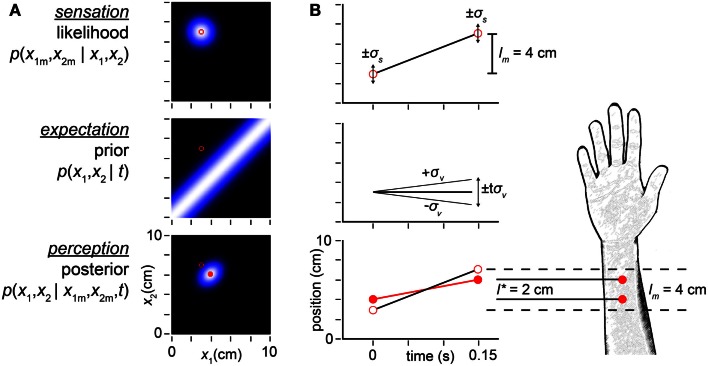
**Bayesian model**. **(A)** The observer’s likelihood function, prior probability density, and posterior probability density in response to taps sensed (i.e., measured by the observer) at positions (*x*_1*m*_, *x*_2*m*_) = (3, 7 cm) (open red circles in all plots). Each pixel in the intensity plots represents a candidate trajectory: a possible tap 1 position and tap 2 position pair (*x*_1_, *x*_2_). Lighter color indicates higher probability (each plot is individually auto-scaled to take advantage of the full brightness range). The measured trajectory length is *l_m_* = *x*_2*m*_ − *x*_1*m*_ = 4 cm. *Top*: the observer’s likelihood function plots the probability of the measured trajectory given each candidate trajectory. The observer understands that a single tap at any location produces a measurement drawn from a Gaussian distribution centered at that location, with standard deviation σ*_s_*; thus, the likelihood function is a two-dimensional Gaussian density centered on the measured trajectory. *Middle*: the observer expects slow movement to occur more commonly; we model this expectation as a Gaussian distribution over trajectory speed, with mean zero and standard deviation, σ*_v_*. Consequently, the observer expects closely spaced taps, and its prior is maximal along the *x*_1_ = *x*_2_ diagonal. *Bottom*: the posterior probability of each trajectory is proportional to the product of its likelihood and prior. The mode of the posterior (filled red circle) is the percept. **(B)** Space-time plots equivalently illustrate the inference process. *Top*: open red circles show measured tap positions (vertical-axis) and times of occurrence (horizontal-axis). Error bars (±1σ_s_) represent the spatial imprecision of the measurements. The slope of the line connecting the taps is the measured trajectory speed: *l_m_* /*t* = 4 cm/0.15 s = 27 cm/s. *Middle*: the observer’s low-speed expectation is represented by the line of slope zero and diagonal lines of slopes ±1σ*_v_* = ±10 cm/s. The distance traversed at speed σ*_v_* in time *t* is *t*σ*_v_* = 1.5 cm. The ascending diagonal line is shallower than the measured velocity: 10 cm/s < 27 cm/s. Equivalently, tσ_v_ = 1.5 cm < *l_m_* = 4 cm. Thus, the measured trajectory violates the observer’s low-speed expectation. *Bottom*: the perceived trajectory (filled red circles and red line) is a compromise between the measured trajectory (open circles, reproduced from top panel) and expectation (middle panel). Each tap has migrated perceptually by 1 cm toward the other, resulting in perceptual length contraction: l* = 2cm < *l_m_* = 4 cm. The perceived trajectory speed is l*/t = 2 cm/0.15 s = 13 cm/s. In both panels, σ*_s_* = 1 cm, σ*_v_* = 10 cm/s, *t* = 0.15 s, *x*_1*m*_ = 3 cm, *x*_2*m*_ = 7 cm.

We begin by considering sequences of two taps, which result in two uncertain spatial measurements (*x*_1*m*_, *x*_2*m*_) and a detected time, *t*, between them^2^. The Bayesian observer (Figure 2) attempts to infer the actual tap positions (*x*_1_, *x*_2_) that produced the measurements (*x*_1*m*_, *x*_2*m*_). We refer to each possible (*x*_1_, *x*_2_) pair as a *candidate* *trajectory*, and to the measured positions (*x*_1*m*_, *x*_2*m*_) as the *measured trajectory*. The Bayesian observer considers both the *likelihood* and the *prior probability* of every candidate trajectory. A trajectory’s likelihood is the probability that the trajectory would give rise to the measured trajectory. The plot of trajectory likelihoods – the likelihood function – is a cloud of uncertainty centered on the measured trajectory (Figure 2A, top). We analogize the likelihood function to a (typically unconscious) *sensation* – a precursor to the conscious percept.

A trajectory’s prior probability is the frequency with which the observer expects the trajectory to occur; this may be the prevalence of the trajectory in nature, which the observer has learned from experience. The plot of prior probabilities – the prior density – represents the observer’s *expectation* regarding trajectory occurrence. Crucially, our Bayesian observer believes that slow trajectories are more common than fast ones. We model this low-speed prior as a Gaussian density over trajectory speed, with mean zero and standard deviation σ*_v_* (the subscript *v* signifies “velocity”). Thus, trajectories in which the two taps are spaced closer together (i.e., lower-speed trajectories) have greater prior probability than those in which the taps are spaced farther apart (Figure 2A, middle).

Using Bayes’ rule, the observer multiplies each trajectory’s likelihood by its prior probability to obtain its posterior (final) probability. In essence, the observer combines *sensation* with *expectation* to achieve *perception*. The mode of the posterior distribution – the most probable trajectory – is the observer’s percept (Figure 2A, bottom). Because of the low-speed prior, the percept underestimates the distance between rapidly presented stimuli. In the example illustrated, whereas the measured tap positions were (3, 7 cm), the percept was (4, 6 cm). The perceived distance between taps (l* = 2 cm) was thus half the measured distance (*l_m_* = 4 cm) (Figures 2A,B).

**Figure 3:**
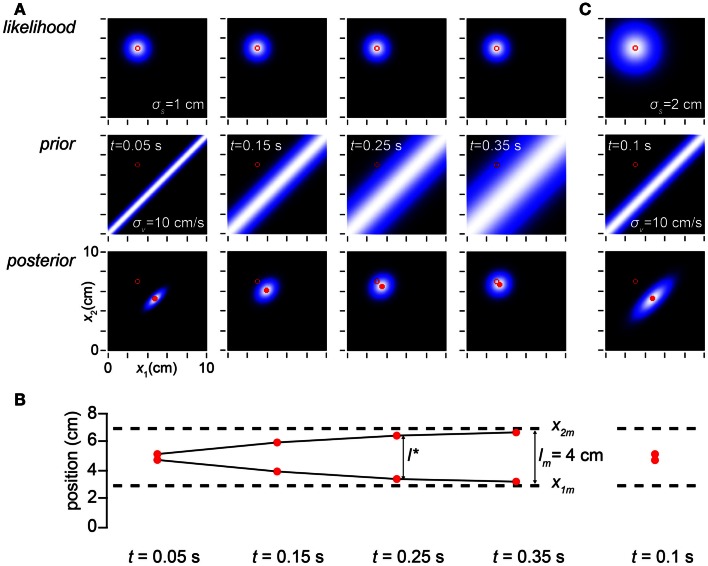
**Time affects space perception**. **(A)** The columns display the observer’s likelihood function, prior probability density, and posterior probability density on four trials in which the measured trajectory (open red circle in all plots) was *x*_1*m*_ = 3 cm, *x*_2*m*_ = 7 cm, and the time, *t*, between taps was (left to right) 0.05, 0.15, 0.25, and 0.35 s. Because the observer has a low-speed expectation, it most strongly expects the taps to fall close together when the time between them is short; thus, the narrowest prior distribution is found in the left column, and the prior distribution widens as *t* increases. The perceived trajectory (mode of the posterior, filled red circle) is pulled closer to the *x*_1_ = *x*_2_ diagonal when the prior is sharper. Therefore, the observer experiences more pronounced length contraction as *t* decreases. Conversely, as *t* increases, length contraction diminishes, and the perceived trajectory asymptotically approaches the measured trajectory (note diminishing distance between filled and open circles in the posterior plots as *t* increases). For all columns, σ*_s_* = 1 cm, σ*_v_* = 10 cm/s. **(B)** The perceived first and second tap positions (filled red circles), corresponding to the mode of each of the posterior plots above, are graphed along with the measured tap positions (dashed lines). The perceived distance between taps asymptotically approaches the measured distance as *t* increases (compare to Figure 1C, lower). **(C)** The amount of perceptual length contraction depends not only on *t* and σ*_v_* but also on σ*_s_*. Here we simulate a trial at *t* = 0.1 s for an observer whose spatial acuity is worse (σ*_s_* = 2 cm) than the observer in **(A)**. Although its posterior density is broader, this observer has the same percept (mode of the posterior) as the observer in **(A)** with *t* = 0.05 s (leftmost column in **A)**. Note that the ratio of σ*_s_* to σ*_v_t* is identical (=2) in the two cases. It is this ratio that determines the amount of perceptual length contraction.

How, exactly, does the time between taps influence perceptual length contraction? This question is answered in Figure 3. Because speed is distance divided by time, the prior probability falls off more sharply with distance when the time between taps is short. While always maximal along the *x*_1_ = *x*_2_ diagonal, the prior widens as ISI increases (Figure 3A, left to right). As a consequence, perceptual length contraction is most pronounced at shorter ISIs; as ISI increases, the perceived distance between taps asymptotically approaches the measured distance (Figure 3B).

**Figure 4:**
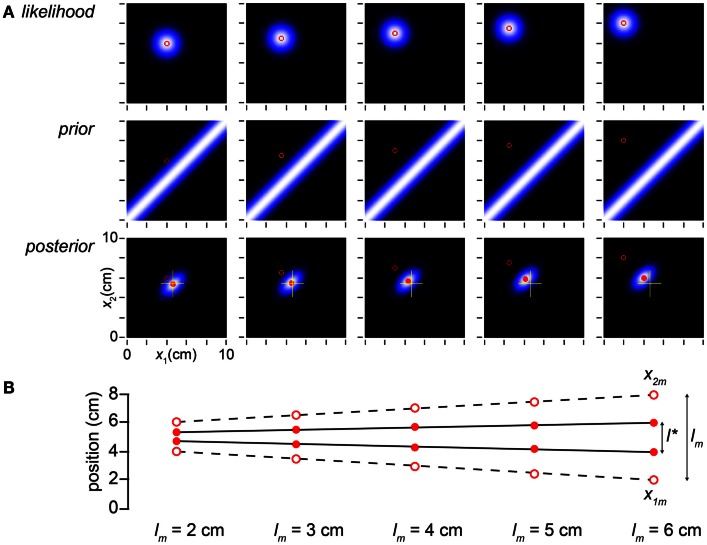
**Perceived distance grows linearly with measured distance**. **(A)** The columns display the observer’s likelihood function, prior probability density, and posterior probability density on five trials, in which the measured distance was progressively increased from 2 to 6 cm while *t* was held constant at 0.1 s. The mode of the posterior (filled red circle) tracks but lags the measured trajectory (open red circle). To facilitate comparison, yellow crosshairs in all posterior plots mark the posterior mode in the leftmost column. **(B)** The measurements, *x*_1*m*_ and *x*_2*m*_, are plotted as open circles; the observer’s percept (mode of the posterior), as filled circles. l* grows linearly with, but consistently underestimates, *l_m_* (compare to Figure 1A, lower). The measurements (*x*_1*m*_, *x*_2*m*_) were, from left to right: (4, 6 cm), (3.5, 6.5 cm), (3, 7 cm), (2.5, 7.5 cm), and (2, 8 cm). In all panels, σ*_s_* = 1 cm, σ*_v_* = 10 cm/s.

We have explained the influence of time on the Bayesian observer’s perception of space, but what of the influence of space itself on space perception? In Figure 4, we find reassuringly that l* varies linearly with *l_m_*, although length contraction ensures that the slope of the relationship is less than one.

## The Perceptual Length Contraction Formula

In the Section “The Bayesian model” in Appendix, we show that the Bayesian observer’s posterior density is a two-dimensional Gaussian distribution. The mode of the posterior reveals a relationship between l* and *l_m_*:

(1) $$l^{*}=\frac{l_{m}}{1+2{\left(\frac{\sigma_{s}}{\sigma_{v}t}\right)}^{2}}$$

Equation 1 is the perceptual length contraction formula, first reported by Goldreich (2007). Notice that, as we have seen, this formula predicts that l* asymptotically approaches *l_m_* in the limit that *t* approaches infinity (Figures 3A,B), that the degree of length contraction is determined by the ratio of σ*_s_* to σ*_v_t* (Figure 3C), and that, at fixed *t*, l* relates linearly to, but underestimates, *l_m_* (Figure 4).

**Figure 5:**
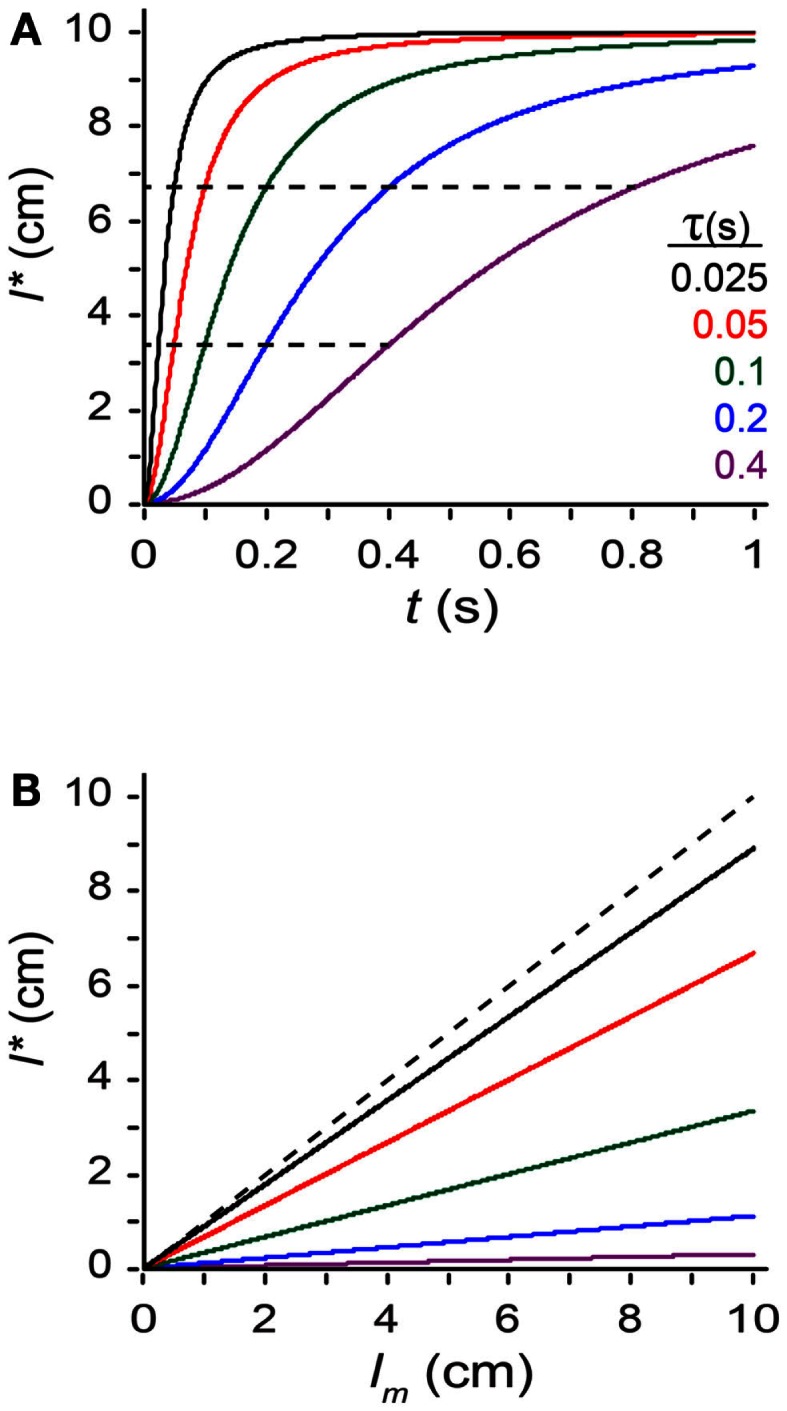
**Exploring the perceptual length contraction formula**. **(A)** Perceived length, l*, plotted against ISI (*t*), for a trajectory of measured length *l_m_* = 10 cm, at five values of the parameter τ (Eq. 2). Perceived length asymptotically approaches measured length as *t* increases. Each curve reaches l* = (1/3) *l_m_* (lower dashed line) when *t* = *τ*, and l* = (2/3) *l_m_* (upper dashed line) when *t* = 2*τ*. **(B)** Perceived length, l*, plotted against measured length, *l_m_*, for a trajectory of *t* = 0.1 s, at five values of τ [color code as in **(A)**]. Perceived length grows linearly with, but underestimates, measured length. Observers with larger τ experience more pronounced length contraction. Dashed diagonal line: l* = *l_m_*.

Because σ*_s_* and σ*_v_* occur only as a ratio in the length contraction formula, it is convenient to rewrite the formula as:

(2) $$l*=\frac{l_{m}}{1+2{\left(\frac{\tau }{t}\right)}^{2}}$$

where tau (*τ*), defined as σ*_s_*/σ*_v_*, has units of time, and is the model’s single free parameter^3^. From Eq. 2 we see that tau is a time constant for space perception. The smaller the value of tau, the more the perceived length increases toward the measured length as inter-stimulus time increases: l* = (1/3) *l_m_* when *t* = *τ*, and l* = (2/3) *l_m_* when *t* = 2*τ* (Figure 5A). Thus, the larger the value of τ, the more susceptible the observer is to perceptual length contraction: for a given *t* and *l_m_*, an observer with a larger τ will perceive a shorter trajectory (Figures 5A,B).

To develop an intuition for these effects of tau, consider that the parameter can be rewritten:

(3) $$\tau =\frac{\sigma_{s}}{\sigma_{v}}=\frac{1/\sigma_{v}}{1/\sigma_{s}}=\frac{\text{strength of low-speed expectation}}{\text{spatial acuity}}$$

Thus, tau reflects the strength of the observer’s low-speed expectation relative to the observer’s spatial acuity. Tau is large in an observer with poor spatial acuity (large σ*_s_*) and a strong expectation for slow movement (small σ*_v_*). This observer places trust in the low-speed expectation; the observer’s perception is considerably length contracted. Tau is small in an observer with excellent spatial acuity (small σ*_s_*) and little expectation regarding movement speed (large σ*_v_*). This observer places trust in the measurement; the observer’s perception is only modestly length contracted.

The perceptual length contraction formula closely fits human data from a variety of experiments (Figure 1; see also Goldreich, 2007 for additional data fits). The fit is particularly satisfying given that the formula has just a single free parameter. The best-fit *τ*-values for the data displayed in Figures 1A–C were 0.21, 0.11, and 0.08 s. The larger *τ* for the Figure 1A fit may reflect the use of electrocutaneous stimuli by Marks et al. (1982), the source of the data plotted in Figure 1A. Electrical pulses tend to be more difficult to localize (larger σ*_s_*) than mechanical taps (Higashiyama and Hayashi, 1993), which were used to generate the data in Figure 1B (Lechelt and Borchert, 1977) and Figure 1C (Kilgard and Merzenich, 1995). Measures of point localization suggest that σ*_s_* is on the order of 1 cm in response to light mechanical stimuli on the forearm (Weinstein, 1968; Martikainen and Pertovaara, 2002; Cody et al., 2008); thus, taking τ = 0.1 s as a nominal value for mechanosensory perception on the forearm, we infer that σ*_v_* is on the order of 10 cm/s.

## Bayesian Perception is Optimal Because It is Beneficially Biased

Before developing our model further, we pause to consider an important conceptual question: we have described the Bayesian observer as achieving an optimal perceptual inference, but we have also shown that the observer consistently underestimates the measured distance between taps. How can an observer be both biased and optimal? This important question applies to any Bayesian observer with a non-uniform prior distribution.

The short answer to the question is that bias is optimal when it accurately reflects the stimulus statistics. In a world in which slow trajectories are more common than fast ones (and, therefore, among trajectories with any given inter-stimulus time, *t*, short lengths are more common than long ones), an observer is justified in perceiving trajectories as shorter than measured. Paradoxically, then, the Bayesian observer is optimal precisely because it is biased.

**Figure 6:**
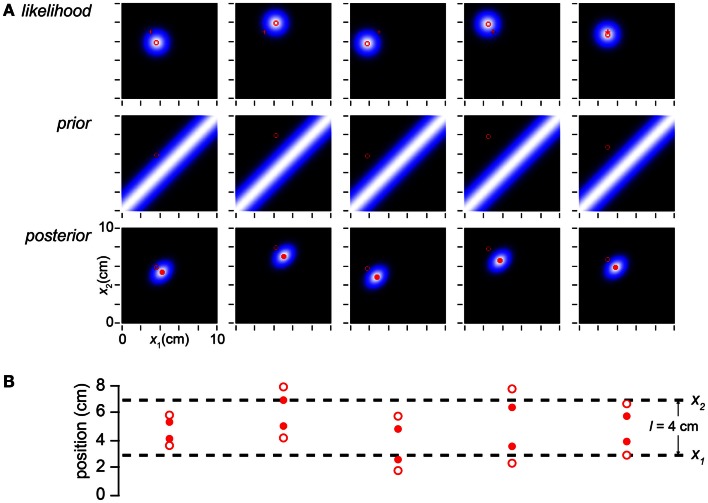
**Measurement noise causes stochastic perception**. **(A)** The columns display the observer’s likelihood function, prior probability distribution, and posterior probability distribution on five trials with the identical stimulus trajectory: *x*_1_ = 3 cm, *x*_2_ = 7 cm, *t* = 0.15 s. Each measured stimulus position was randomly sampled from the true location; thus, the measured trajectory (*x*_1*m*_, *x*_2*m*_; open red circle) bounces randomly from trial to trial around the fixed true value (3, 7 cm; red cross). Because the likelihood function is centered on the measurement, it too bounces. Consequently, the observer’s percept (mode of the posterior, filled red circle) varies stochastically from trial to trial. **(B)** The measured tap positions (open circles) and perceived tap positions (mode of posterior, filled red circles) on each trial, compared to the actual tap positions (dashed lines). On every trial, the perceived trajectory length (l*, distance between filled circles) underestimates the measured length (*l_m_*, distance between open circles); the perceived trajectory length therefore on average underestimates the actual trajectory length (*l*).

To understand this thoroughly, we must appreciate the consequences of both measurement and stimulus variability. In Figures 2–5 we artificially specified (*x*_1*m*_, *x*_2*m*_). In a laboratory experiment, however, the investigator can control only the stimulus, not the measurements. As explained, we conceive of each measured tap location as sampled from a Gaussian distribution of standard deviation σ*_s_*, centered on the actual tap location. Thus, if the skin is stimulated repeatedly with the identical trajectory, the measurement and consequently the percept will vary stochastically from trial to trial (Figure 6).

**Figure 7:**
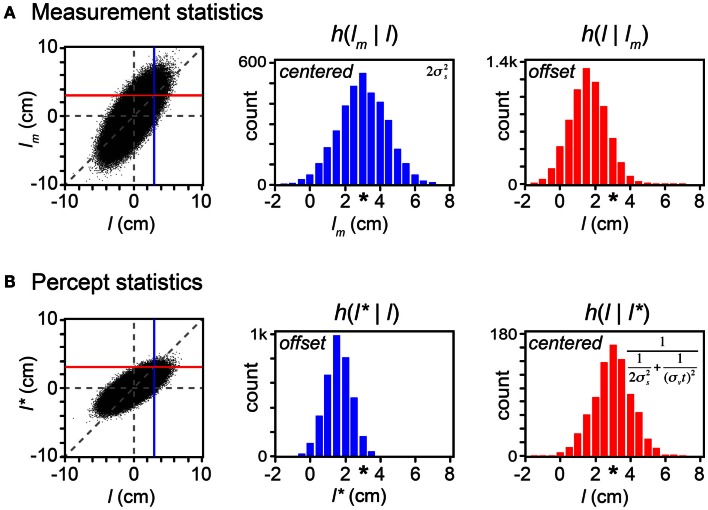
**Bayesian perception is optimal because it is biased**. On each of 1 million trials, a first tap position (*x*_1_) was drawn from a uniform distribution, and a second tap position (*x*_2_) was drawn from a Gaussian distribution centered on the first tap position, with standard deviation *t*σ*_v_* = 1.5 cm (i.e., σ*_v_* = 10 cm/s, *t* = 0.15 s; see Eq. A8 in Appendix). Measured positions, *x*_1*m*_ and *x*_2*m*_, were then drawn independently from Gaussian distributions of standard deviation σ*_s_* = 1 cm, centered on the corresponding tap positions (*x*_1_ and *x*_2_). **(A)** *Left*: scatterplot of measured trajectory length (*l_m_* = *x*_2*m*_ −  *x*_1*m*_) against actual trajectory length (*l* = *x*_2_ − *x*_1_) for each of the trials (dots); negative lengths indicate trajectories in which *x*_2_ < *x*_1_. Dashed vertical and horizontal lines: *l* = 0 and *l_m_* = 0. Diagonal dashed line: *l_m_* = *l*. Vertical blue line: *l* = 3 cm. Horizontal red line: *l_m_* = 3 cm. *Center*: histogram (h) of *l_m_* values that occurred when *l* was between 2.95 and 3.05 cm (i.e., *l_m_* samples that fell along the blue vertical line in the scatterplot). The histogram is a Gaussian distribution centered at *l_m_* = 3 cm (asterisk). *Right*: histogram of *l* values of trajectories that gave rise to *l_m_* between 2.95 and 3.05 cm (i.e., *l* samples that fell along the red horizontal line in the scatterplot). The histogram represents the observer’s posterior density over *l*. It is a Gaussian distribution centered at *l* = 1.6 cm, not 3 cm (asterisk). **(B)** Left, center, and right panels as in **(A)**, but for l* rather than *l_m_*. *Center*: l* is a biased estimator. *Right*: on trials in which the observer perceived l* = 3 cm, the true trajectory length averaged 3 cm. Because the perceived length is a deterministic function of the measurement, this histogram has the same variance as the posterior density over *l*. Inset formulas in **(A)** *center* and **(B)** *right* show the variances of these histograms (See “One-dimensional reductions” in Appendix). These are equal to the mean-squared error between each estimator and the true length.

By incorporating measurement variability, the simulation shown in Figure 6 is a more realistic representation of a laboratory experiment than are the simulations shown in the earlier Figures. Crucially for our understanding of the paradox of bias and optimality, however, Figure 6 would be an unrealistic portrayal of the Bayesian observer’s experience in the real-world. In the real-world, not only the measurements but also the trajectories themselves are drawn from a distribution. In Figure 7, we more closely simulate what we envision to be real-world tactile experience. The figure plots the lengths of one million trajectories sampled from a zero-mean velocity distribution (for clarity of illustration, all with *t* = 0.15 s), from each of which spatial measurements were sampled and processed into a percept.

A comparison of the statistics of the measured length, *l_m_* (Figure 7A) with those of the perceived length, l* (Figure 7B) reveals that, although the observer’s perception is biased, it is more accurate than the measurement. In fact, the observer’s perception is optimal precisely because it is biased. To understand why, consider that the majority of these real-world trajectories have very short lengths (*l* close to zero). Because short trajectories are more common, any measured length, *l_m_*, most often originates from a trajectory of shorter true length, *l*. The Bayesian observer’s percept is biased by the prior to take this crucial knowledge into account; consequently, over the course of many trials, the percept more closely reflects the true stimulus than the measurement does. This is indicated by the smaller vertical scatter of the percept (Figure 7B, left) than of the measurement (Figure 7A, left) around the diagonal line.

Further inspection of the scatterplot in Figure 7A reveals that, for any true trajectory length, *l*, the measurement, *l_m_*, occurs with equal frequency above and below the diagonal line. Thus, the histogram of *l_m_* samples is centered on *l* (Figure 7A, center). For this reason, the measured length is termed an “unbiased estimator” of the true length. Despite this lofty denomination, however, it is clear from the same scatterplot that for any magnitude *l_m_* other than 0, the distribution of true lengths has a smaller average magnitude (when *l_m_* > 0, *l* tends to lie to the left of the diagonal line; when *l_m_* < 0, *l* tends to lie to the right of the diagonal line). Thus, *l_m_* is an inaccurate estimator in the sense that the stimuli that result in a particular *l_m_* are on average offset from that *l_m_* (Figure 7A, right). If an observer were to report *l_m_* as the estimate of trajectory length, the observer would be found to systematically report trajectories as being longer than they actually are.

Figure 7B shows that the statistics of the perceived length, l*, are opposite in character to those of the measured length. For any true trajectory length, *l*, the perceived length, l*, systematically underestimates the magnitude of *l* (Figure 7B, left and center). Thus, the perceived length is termed a “biased estimator.” This bias is beneficial, however: because of it, at any l*, the distribution of true lengths is centered on a mean of l* (the values of *l* are symmetrically distributed around the diagonal line in the scatterplot). Thus, l* is an accurate estimator in the sense that the stimuli that result in a particular l* indeed on average have length equal to that l* (Figure 7B, right). The observer’s report of l* can be trusted as accurately reflecting, on average, the true trajectory length. Importantly, the variance of *l* given l* (Figure 7B, right) is smaller than the variance of *l_m_* given *l* (Figure 7A, center). This again reveals that the percept is more accurate than the measurement.

## Selective Spatial Attention Shifts the Perceived Trajectory

Up to this point, we have assumed that the observer’s spatial uncertainty, σ*_s_*, is uniform within the tested area (σ*_s_* will, of course, differ between body areas, such as forearm and finger). However, spatial attention is associated with cortical receptive field recruitment and sharpening within the attended area (Anton-Erxleben and Carrasco, 2013). Thus, if an observer were to focus attention preferentially on one location, we might expect σ*_s_* to decrease there while plausibly increasing at unattended locations. Indeed, on the arm, the spatial error of localization decreases by as much as 30% when attention is directed to the stimulated skin region (Moore et al., 1999; O’Boyle et al., 2001).

**Figure 8:**
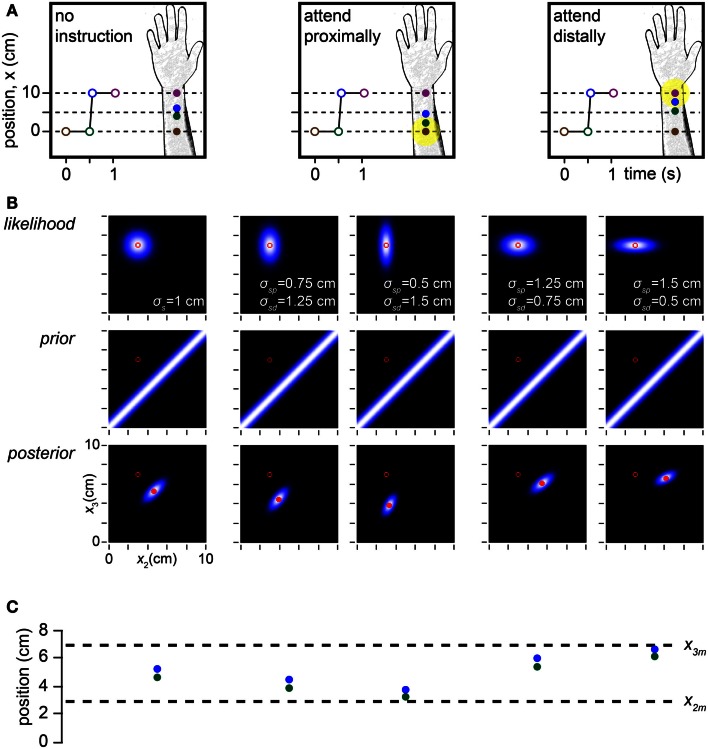
**Modeling the effects of spatial attention**. **(A)** Depiction of a cutaneous rabbit illusion experiment reported by Kilgard and Merzenich (1995). Participants either received no specific instruction or were instructed to direct their attention (yellow highlight) toward the proximal or distal forearm. The investigators found that in the directed attention conditions, the perceived positions of tap 2 (green) and tap 3 (blue) were shifted toward the attended location (forearm sketches). **(B)** In the Bayesian observer, a reduction in σ*_s_* at the attended relative to the unattended location reproduces the perceptual shift reported by Kilgard and Merzenich (1995). *Left panel*: the Bayesian observer’s likelihood function, prior and posterior density when σ*_s_* does not vary with location, simulating the no-instruction condition in **(A)**. In this case, the perceived and measured trajectory midpoints coincide. *Center two panels*: effect of σ*_sp_* < σ*_sd_*, where the subscripts *p* and *d* refer to the proximal and distal arm areas. The greater the reduction of σ*_sp_* relative to σ*_sd_*, the more the perceived trajectory migrates proximally toward the tap 2 measurement. *Right two panels*: effect of σ*_sd_* < σ*_sp_*. The greater the reduction of σ*_sd_* relative to σ*_sp_*, the more the perceived trajectory migrates distally toward the tap 3 measurement. For all plots in **(B)**, the measurements (*x*_2*m*_, *x*_3*m*_) were (3, 7 cm), the time between taps 2 and 3 was 0.06 s, and σ*_v_* was 10 cm/s. **(C)** The perceived (mode of posterior) tap 2 and 3 positions (green and blue circles) for each of the five conditions in **(B)** directly above, compared to the measured tap positions (dashed lines).

If spatial acuity is modulated by selective attention, how might length contraction percepts be affected? In a cutaneous rabbit experiment, Kilgard and Merzenich (1995) found that when participants were not asked to focus their attention to any particular area of the arm, the midpoints of the perceived and actual trajectories tended to coincide (Figure 8A, left). In contrast, when participants were instructed to direct their attention either distally or proximally, the midpoint of the perceived trajectory shifted toward the attended location (Figure 8A, center, right). This occurred because the tap within the attended skin area migrated less perceptually than did the tap within the unattended area, an effect confirmed by Flach and Haggard (2006).

The Bayesian observer replicates this attention effect: when σ*_s_* decreases in one skin area relative to the other, the perceived trajectory midpoint shifts toward the attended location (Figures 8B,C). The relatively precise measurement of the “attended tap” impedes its perceptual migration, while the relatively imprecise measurement of the “unattended tap” facilitates its perceptual migration. In this situation, length contraction is accomplished primarily by the perceptual displacement of the unattended tap.

In the Section “Generalization to inhomogeneous spatial uncertainty” in Appendix, we derive a generalization of the length contraction formula that incorporates separate σ_*s*1_ and σ_*s*2_ values representing spatial uncertainty around the two tap locations. In the general equation, the single spatial uncertainty, σ_*s*_, of Eq. 1 is replaced by the root-mean-square uncertainty at the two locations, σ_rms_:

(4) $$l^{*}=\frac{l_{m}}{1+2{\left(\frac{\sigma_{s\left(\text{rms}\right)}}{\sigma_{v}t}\right)}^{2}}=\frac{l_{m}}{1+\frac{\sigma_{s1}^{2}+\sigma_{s2}^{2}}{{\left(\sigma_{v}t\right)}^{2}}}$$

We show further that the shift, Δ_midpt_, in the perceived trajectory midpoint away from the measured trajectory midpoint is:

(5) $$\Delta_{\text{midpt}}=\frac{l_{m}}{2}\left(\frac{\sigma_{s1}^{2}-\sigma_{s2}^{2}}{{\left(\sigma_{v}t\right)}^{2}+\sigma_{s1}^{2}+\sigma_{s2}^{2}}\right)$$

## The Predictive-postdictive Formulation

The rabbit illusion is often described as providing compelling evidence for perceptual postdiction, a process whereby the perception of an earlier event is modified by the occurrence of a later one. Postdiction is indeed an attractive explanation for the perceptual migration of tap 2 toward the location of tap 3 in the rabbit illusion (Figure 1C). As shown by Kilgard and Merzenich (1995), tap 3 also migrates perceptually toward the location of tap 2 (Figure 1C). Therefore, prediction apparently is also at play: the perception of a later event (tap 3) depends upon an earlier one (tap 2).

In light of these considerations, it may seem surprising that our Bayesian observer replicates length contraction illusions without explicitly representing either pre- or postdictive inference. How is this possible? The answer is that pre- and postdiction are implicitly embedded in the model via the action of the low-speed prior. The low-speed prior transforms the observer’s likelihood function into a posterior density by pulling the observer’s perception of each tap position toward the measured position of the other (Figure 2).

**Figure 9:**
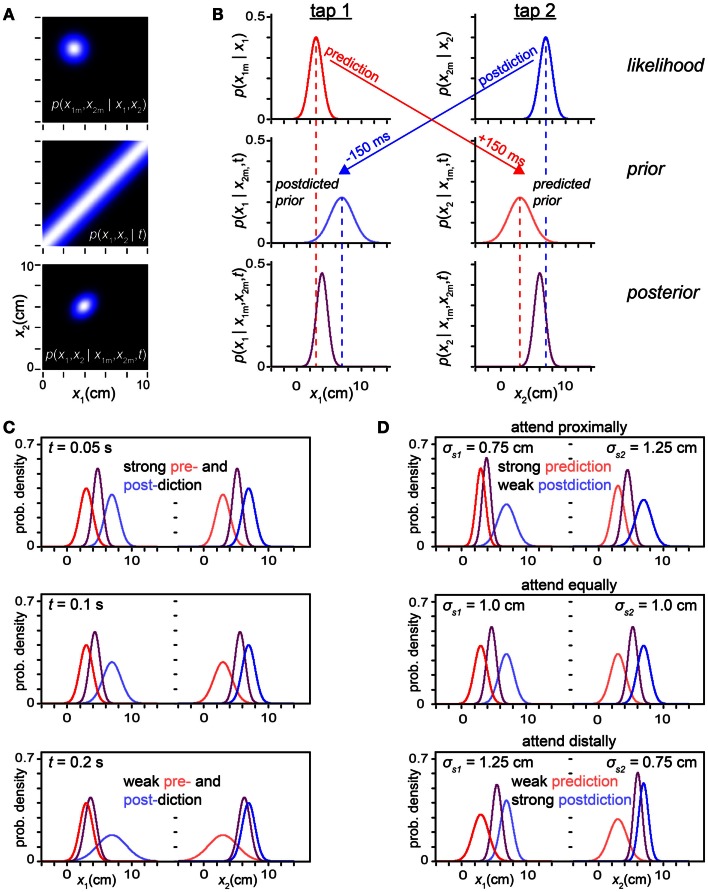
**Prediction-postdiction formulation**. **(A)** The observer’s two-dimensional joint (*x*_1_, *x*_2_) likelihood function, prior and posterior densities. The measured trajectory was *x*_1*m*_ = 3 cm, *x*_2*m*_ = 7 cm, with *t* = 0.15 s. The observer settings were σ*_s_* = 1 cm, σ*_v_* = 10 cm/s. **(B)** The inference process in **(A)** reformulated as a series of one-dimensional inferences regarding *x*_1_ and *x*_2_ individually. *Top left*: the tap 1 likelihood function (red), *p*(*x*_1*m*_ | *x*_1_), is centered on *x*_1*m*_. Because of its low-speed expectation, the observer predicts (red arrow) that the most probable position for a future tap 2 will also be 3 cm. *Middle right*: the observer’s *predicted prior* over tap 2 (light red) represents its belief concerning the position of tap 2, projected 150 ms forward in time from the occurrence of tap 1. *Top right*: the observer’s tap 2 likelihood function (blue), *p*(*x*_2*m*_ | *x*_2_), is centered on *x*_2*m*_. Because of its low-speed expectation, the observer postdicts (blue arrow) that the most probable position for the preceding tap 1 was also 7 cm. *Middle left*: the observer’s *postdicted prior* over tap 1 (light blue) represents its belief concerning the position of tap 1, projected 150 ms backward in time from the occurrence of tap 2. *Left column*: using Bayes’ theorem, the observer multiplies the tap 1 likelihood function (red) by the tap 1 postdicted prior (light blue) to obtain the tap 1 posterior (purple). *Right column*: similarly, the observer multiplies the tap 2 likelihood function (blue) by the tap 2 predicted prior (light red) to obtain the tap 2 posterior (purple). **(C)** Individual tap likelihoods, priors, and posteriors graphed with the same color scheme as in **(B)**, for three trajectories of progressively increasing ISI. At *t* = 0.05 s, pre- and postdiction both result in relatively sharp priors that exert a strong influence over the percept (mode of the posterior). As *t* is increased, the pre- and postdicted priors become lower and broader: pre- and postdiction become increasingly uncertain with the passage of time. The priors thus exert diminishing influence, and the percept approaches the measurement (compare to Figure 3A). For all panels in **(C)**, σ*_s_* = 1 cm, σ*_v_* = 10 cm/s. **(D)** Effect of directed spatial attention, as in Figure 8. *Top*: a reduction in σ_*s*1_ sharpens the tap 1 likelihood function, increasing the strength of prediction (note sharp predicted prior over tap 2), while an increase in σ_*s*2_ broadens the tap 2 likelihood function, decreasing the strength of postdiction (note broad postdicted prior over tap 1). *Middle*: when σ_*s*1_ = σ_*s*2_, pre- and postdiction have equal strength. *Bottom*: reduction in σ_*s*2_ relative to σ_*s*1_ results in effects opposite those seen in the top panel. For all panels in **(D)**, *t* = 0.06 s, σ*_v_* = 10 cm/s.

We can reveal the pre- and postdiction hidden in the Bayesian observer by decomposing the model’s two-dimensional (*x*_1_, *x*_2_) calculations (Figure 9A) into a series of one-dimensional inferences regarding each tap’s position individually (Figure 9B). Using its low-speed expectation, the observer can from the first tap’s likelihood function predict a probability distribution over the position of the subsequent, second, tap, and from the second tap’s likelihood function postdict a probability distribution over the position of the previous, first, tap (arrows in Figure 9B). We call these two distributions the *predicted prior* and *postdicted prior* densities^4^.

Next, the observer simply multiplies each tap’s likelihood function by that tap’s prior to obtain the posterior density over the tap’s position. We show in the Sections “One-dimensional reductions” and “The prediction-postdiction formulation” in Appendix that the posteriors so obtained are identical to those that would result from extracting one-dimensional distributions from the joint (*x*_1_, *x*_2_) posterior: if the joint posterior (Figure 9A, bottom) were marginalized (i.e., integrated) vertically, it would yield the posterior over *x*_1_ shown in Figure 9B, bottom left; if integrated horizontally, it would yield the posterior over *x*_2_ shown in Figure 9B, bottom right.

In the Section “The prediction-postdiction formulation” in Appendix, we show that the predicted and postdicted priors are Gaussian densities, and that their means and variances are:

(6) $$\begin{array}{cccc} \mu_{\text{pre}} & =x_{1m} & \mu_{\text{post}}\; & \;=x_{2m}\\ \sigma_{\text{pre}}^{2} & =\sigma_{s1}^{2}+{\left(\sigma_{v}t\right)}^{2} & \sigma_{\text{post}}^{2}\; & \;=\sigma_{s2}^{2}+{\left(\sigma_{v}t\right)}^{2} \end{array}$$

Equations 6 show that the prior density over each tap’s position is centered on the measurement of the other tap, reflecting the observer’s low-speed expectation (the most probable speed being zero). The variance of each prior density reflects the observer’s uncertainty regarding the other tap’s measurement (σ_*s*1_ or σ_*s*2_) and the observer’s prior uncertainty regarding trajectory speed (σ*_v_*), which translates into an increasing uncertainty regarding the distance traversed as the elapsed time, *t*, increases (σ*_v_t*). Thus, perceptual length contraction diminishes with increasing *t* (Figure 9C), as shown previously (Figures 3 and 5A).

Figure 9D shows that the predictive-postdictive formulation accurately reproduces the effects of directed spatial attention, previously explored in Figure 8. When attention is directed around the location of the first tap (σ_*s*1_ < σ_*s*2_), the predicted prior is sharper than the postdicted prior (σ^2^_pre_ < σ^2^_post_). Consequently, prediction exerts a dominant influence, perceptually displacing the second tap asymmetrically toward the first (Figure 9D, top). When attention is directed around the location of the second tap (σ_*s*2_ < σ_*s*1_), the postdicted prior is sharper (σ^2^_post_ < σ^2^_pre_). In this case, postdiction dominates, perceptually displacing the first tap asymmetrically toward the second (Figure 9D, bottom).

## The Perception of Multi-Tap Sequences

Up to this point, we have modeled the perception of two-tap trajectories^5^. How might a Bayesian observer handle multi-tap sequences, delivered conceivably to any number of skin sites? An observer could apply a low-speed prior independently to the movement between each tap and the next one. Alternatively, an observer might apply a low-speed prior to the first tap pair of the sequence, but thereafter incorporate an expectation that the velocity of each pair be similar to that of the preceding pair: a low-acceleration prior (See “Multi-tap perception” in Appendix).

**Figure 10:**
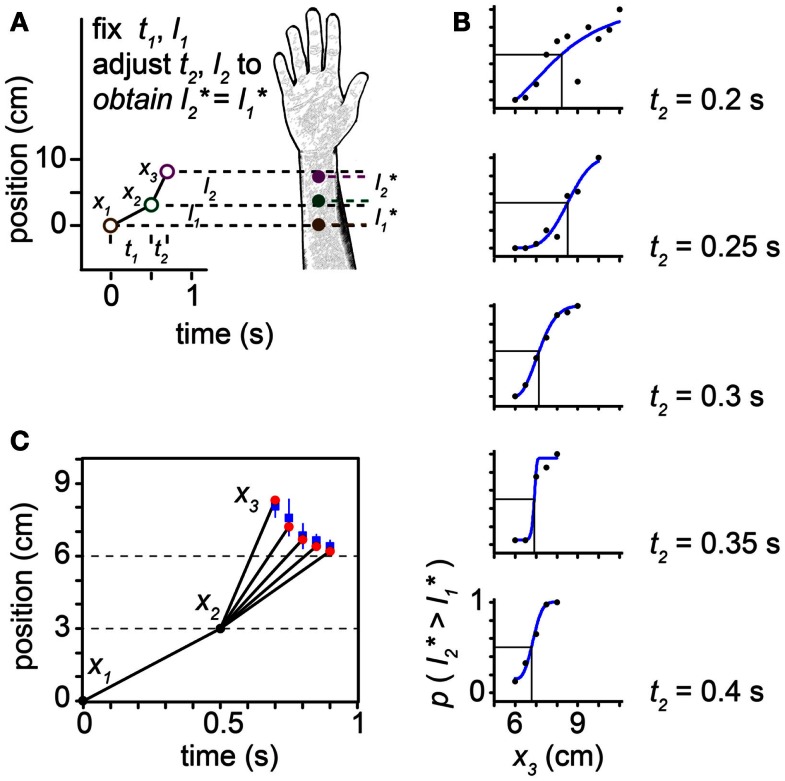
**The tau effect**. **(A)** Three taps to the arm, at positions *x*_1_ = 0 cm, *x*_2_ = 3 cm, and *x*_3_ (variable), define two spatial intervals, *l*_1_ = 3 cm and *l*_2_ (variable), and two temporal intervals, *t*_1_ = 0.5 s and *t*_2_ (variable). Because *t*_2_ < *t*_1_, at some *l*_2_ > *l*_1_ the two intervals will be perceived to be of equal length (*l*_2_* = *l*_1_*). **(B)** At each of five *t*_2_ settings (identified at right of plots), Helson and King (1931) progressively increased *l*_2_ by shifting *x*_3_ along the arm in 0.5-cm increments. On each trial, the participant reported whether the second spatial interval was perceived to be shorter than, equal to, or longer than the first interval. To accurately estimate each participant’s point of subjective equality (PSE), we transformed these data into a two-alternative forced-choice format by distributing the participant’s “equal” responses evenly to the “shorter” and “longer” response categories. We then fit each participant’s transformed data (proportion “*l*_2_ is longer” responses) at each *t*_2_ setting with a Weibull psychometric function (blue curves). Each psychometric function provides a PSE (vertical line): the *x*_3_ at which the psychometric function intersected 0.5 (horizontal line), indicating that *l*_2_* = *l*_1_*. The PSE shifted progressively to the left as *t*_2_ was increased (note: when *x*_3_ = 6 cm, *l*_2_ actually does equal *l*_1_). The transformed data shown are from one participant (“Observer C”) in Helson and King (1931). **(C)** Trajectories for which *l*_2_* = *l*_1_*. Blue points: mean *x*_3_ that resulted in *l*_2_* = *l*_1_* among the six participants tested by Helson and King (1931), at each of the five *t*_2_ settings. Blue lines: ±1 SD. Red points: best-fit performance of the Bayesian low-speed observer (τ = 0.10 s).

**Figure 11:**
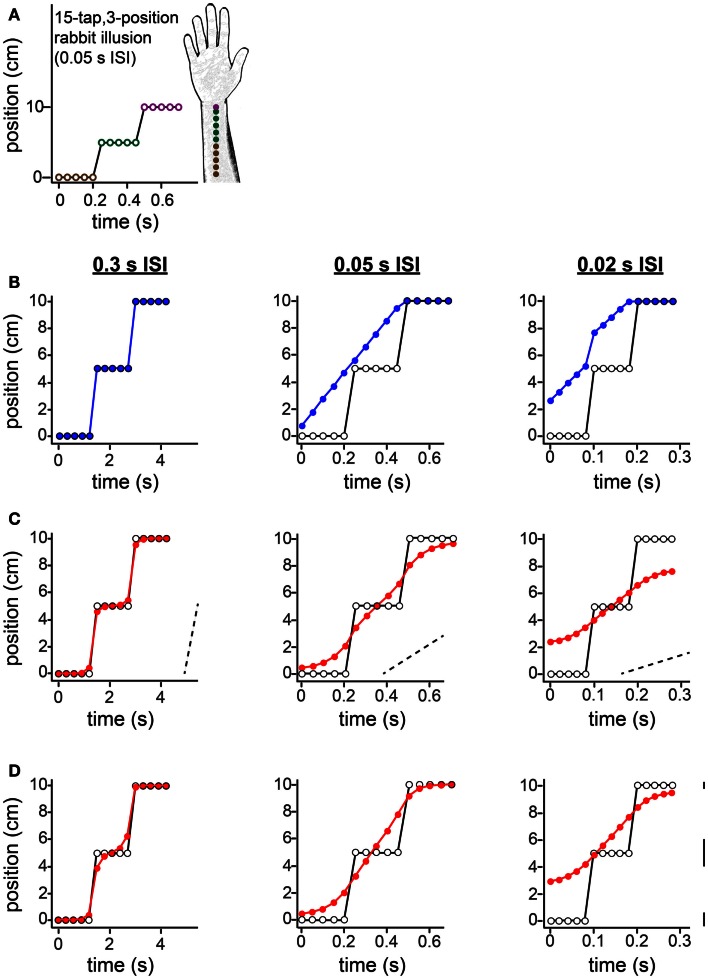
**The 15-tap rabbit illusion**. **(A)** Geldard (1982) delivered five taps at each of three locations along the arm. When ISI between successive taps was 0.05 s, participants reported perceiving a linear spatial progression of taps 1 through 10 (forearm sketch). **(B)** The same spatial sequence shown in **(A)**, at three different ISIs, resulted in distinct percepts (Geldard, 1982). *Left*: at 0.3 s ISI, perception was veridical. *Center*: at 0.05 s ISI, perception was as shown in **(A)**. *Right*: at 0.02 s ISI, the taps were perceived to begin at a position between 2 and 3 cm along the arm, and to advance in a non-linear spatial progression. Open circles: true tap positions; blue points: human perceptual report. **(C)** The Bayesian low-speed observer’s perception with a standard setting of τ = 0.10 s (e.g., σ_*s*_ = 1 cm, σ*_v_* = 10 cm/s) shows much similarity to participants’ subjective reports. Open circles: true tap positions; red points: Bayesian observer’s perception (mode of the posterior). Dashed slanted lines have slope 10 cm/s (i.e., 1σ*_v_*). Note that the two rapid jumps in the true trajectory (from tap 5 to tap 6, and from tap 10 to tap 11) occur at a speed much greater than σ*_v_* when the ISI is 0.05 s (*center*) or 0.02 s (*right*); thus, perceptual length contraction occurs in these cases. In contrast, at an ISI of 0.3 s (*left*), the trajectory does not strongly violate the observer’s low-speed expectation; thus, perception is nearly veridical. **(D)** The Bayesian low-speed observer’s perception can be made even closer to human reports if the value of σ_*s*_ varies along the arm. The observer’s percept at each ISI is shown for σ_*s*_ = 1, 2, and 0.5 cm around the proximal, middle, and distal arm regions, respectively. Line segments at right have length equal to 1σ_*s*_ at each location. The value of σ*_v_* was fixed at 10 cm/s.

**Figure 12:**
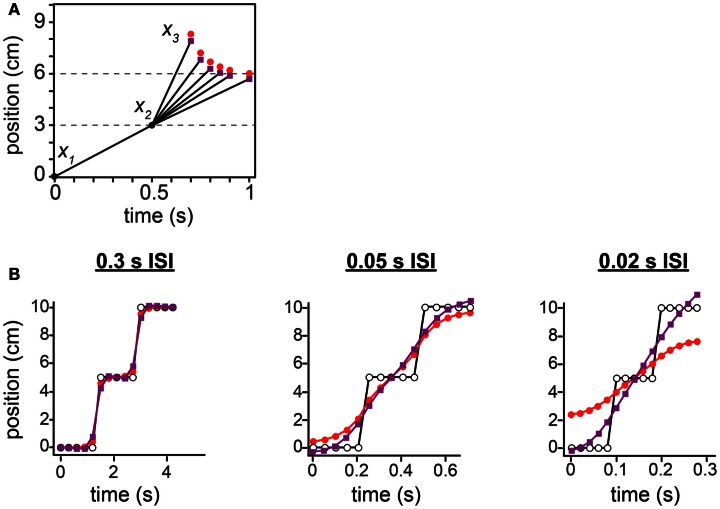
**Comparison between the low-speed-prior and low-acceleration-prior observers**. **(A)** The tau effect. Red points: low-speed-prior observer’s performance, reproduced from Figure 10C, and extended to 1 s on the *x*-axis. Purple points: low-acceleration-prior observer’s performance. **(B)** The 15-tap rabbit. Red points: low-speed-prior observer’s performance, reproduced from Figure 11B. Purple points: low-acceleration-prior observer’s performance. For both observers in **(A)** and **(B)**, τ was set to 0.10 s (i.e., σ_*s*_ = 1 cm, σ*_v_* = 10 cm/s).

Here, we test each of these Bayesian observers with multi-tap sequences that produce illusions in humans. We consider two well-known illusions. The first is the tau effect, so-named by Helson (1930) and subsequently described in elegant detail by Helson and King (1931). The second is a multi-tap rabbit, characterized in a delightful paper by Geldard (1982). In Figures 10 and 11, we show that the observer with a low-speed prior produces good fits to the human perceptual data; in Figure 12, we show that the observer with a low-acceleration prior does not.

In the tau effect experiment, taps at three skin positions define two spatial and two temporal intervals (Figure 10). Helson and King (1931) reported that, when *t*_2_ = *t*_1_ and *l*_2_ = *l*_1_, the participants perceived the two lengths as equal: $l_{2}^{*}\;=\;l_{1}^{*}$. As *t*_2_ was progressively reduced, however, tap 3 had to be located progressively farther down the arm (i.e., *l*_2_ had to be progressively increased) in order to make $l_{2}^{*}$ equal $l_{1}^{*}$ (Figures 10B,C). The best-fit of our low-speed-prior observer to the average of the human data occurred at τ = 0.10 s. The Bayesian observer closely replicated the space-time curve characterizing human perception (Figure 10C).

In the 15-tap rabbit experiment, five taps are delivered consecutively at each of three positions along the arm (Figure 11). Geldard (1982) found that when the time between consecutive taps was 0.05 s, participants perceived the first 10 taps in the sequence as hopping at an approximately uniform rate up the arm, each tap displaced by a constant spatial increment from the preceding one (Figures 11A,B, center). At an ISI of 0.3 s, perception was reportedly veridical (Figure 11B, left). At an ISI of 0.02 s, the perceived sequence began partway up the arm and traced a non-linear, somewhat sigmoidal path (Figure 11B, right).

The low-speed-prior observer’s perception with τ = 0.10 s agrees qualitatively with the perception of human participants (Figure 11C). To understand why, first note that, at an ISI of 0.05 s (Figure 11C, center) or 0.02 s (Figure 11C, right), the rapid jumps in the stimulus sequence are in clear violation of the observer’s low-speed expectation (see diagonal dotted lines with slope σ*_v_*). Consequently, perceptual length contraction occurs for those tap pairs: the perceived distance between taps 5 and 6, and between taps 10 and 11, is considerably smaller than the actual distance. Now, what causes the progressive perceptual displacement of the many taps that are, in reality, at the same position? Interestingly, each jump in the actual stimulus sequence results in a chain reaction that propagates, with diminishing strength, to more distant taps. The rapid jump from tap 5 to tap 6 induces perceptual length contraction that pulls tap 5 considerably upward in the plot (and tap 6 downward). This places perceived distance between taps 4 and 5, which given the short ISI is sufficient to violate the observer’s low-speed expectation as applied to that tap pair. Consequently, taps 4 and 5 are perceptually attracted, resulting in some upward perceptual displacement of tap 4, placing perceptual distance between it and tap 3, and so on.

How would perception of the 15-tap sequence change if the observer were to direct its spatial attention unequally along the arm? To explore this question, in Figure 11D we have plotted the low-speed-prior observer’s perception under conditions of “standard” attention to the proximal arm (σ_*s*_ = 1 cm), directed attention to the distal arm (σ_*s*_ = 0.5 cm), and relative inattention (σ_*s*_ = 2 cm) to the area in-between. Comparison of Figures 11D,C indicates that adjustment to spatial attention affects perception in ways that depend upon ISI. For the particular values of σ_*s*_ used in this example, perception of the 0.3 s ISI sequence remains nearly veridical (Figure 11D, left), whereas perception of the 0.05 s ISI sequence to some extent (center), and of the 0.02 s ISI sequence to a greater extent (right), are shifted upwards in the plots. The result is that the observer’s perception even more closely resembles that of the human participants reported by Geldard (1982).

Unlike the low-speed-prior observer, the low-acceleration-prior observer distinctly fails to match human perception (Figure 12). In the tau effect scenario, a discordant feature of the low-acceleration-prior observer is that, when *t*_2_ = *t*_1_ and *l*_2_ = *l*_1_, the observer fails to perceive the lengths as equal, instead perceiving *l*_2_* > *l*_1_*. This perceptual asymmetry occurs because only the first segment of the trajectory is subject to a low-speed prior. Thus, when *t*_2_ = *t*_1_, *l*_2_ must be made shorter than *l*_1_ in order to be perceived as equal. Consequently, in our simulation of Helson and King (1931) using the low-acceleration-prior-observer, *x*_3_ fails to converge to 6 cm as the tap 3 time approaches 1 s (Figure 12A, purple points). The performance of the low-speed-prior observer, in contrast, does converge as expected (red points).

In the 15-tap rabbit experiment, at 0.05 s ISI and more markedly at 0.02 s ISI, the low-acceleration-prior observer perceives the trajectory to start below the actual tap 1 location and to end above the actual tap 15 location: the perceived trajectory is longer than the actual trajectory (Figure 12B, purple points). This is incompatible with human perceptual report, and opposite to the perception of the low-speed-prior observer (red points). The perceptual undershoot and overshoot occur because the rapid jumps in the actual stimulus sequence extend perceptually in both directions at nearly constant velocity, in keeping with the observer’s low-acceleration expectation.

## Discussion

### Perceptual length contraction as Bayesian inference

Length contraction illusions have long fascinated and puzzled investigators. The tactile tau effect was first reported almost 100 years ago (Gelb, 1914). It was later named and investigated in detail in the early 1930s (Helson, 1930; Helson and King, 1931). The best-known length contraction illusion, the cutaneous rabbit, was discovered serendipitously some 40 years later, when Geldard and colleagues, intending to study the tau effect, mistakenly produced a stimulus pattern similar to the rapid sequences shown in Figure 11B (Geldard and Sherrick, 1972; Geldard, 1982). The resulting perception of taps hopping up the arm led a surprised observer to exclaim “who let the rabbit loose?” (Geldard, 1982). Over the years, investigators have proposed creative explanations – geometrical, mathematical, and neural – for these and related illusions (Jones and Huang, 1982; Brigner, 1988; Wiemer et al., 2000; Grush, 2005; Flach and Haggard, 2006).

The Bayesian observer model expounded here provides a concise and coherent explanation for the tau effect, the cutaneous rabbit, and related spatiotemporal illusions. Elapsed time influences the perception of traversed space because the observer expects objects to move slowly. In its simplest form, the model contains a single free parameter, tau: a time constant for space perception (Eqs 2 and 3). While much research remains to be done, we are encouraged by the close fit of the model to human perceptual data. Because a single model replicates the tau effect (Figure 10), the rabbit (Figures 1C and 11), and other spatiotemporal illusions (Figures 1A,B; see also Goldreich, 2007), we suggest that these illusions are manifestations of a single perceptual assumption: a low-speed prior. Our confidence in this suggestion is strengthened by the finding that a single value of the tau parameter (∼0.1 s) provides good fits to perception on the forearm as measured in experiments using different paradigms and carried out by multiple laboratories.

A central feature of Bayesian perceptual models is that they consider multiple hypotheses – in our case, candidate trajectories. The idea that the brain perceives by evaluating candidates is consistent with the “multiple drafts” theory of Dennett and Kinsbourne (1992). These authors propose that, confronted with stimuli such as those depicted in Figure 11, the brain favors a distributed sequence of taps as the most “parsimonious” interpretation. This suggestion is compatible with our model if one equates parsimony with posterior probability. However, Dennett and Kinsbourne (1992) do not explain on what grounds an observer judges a particular interpretation to be the most parsimonious, nor do they explain why the percept changes as a function of ISI.

Bayesian perceptual models make precise, quantitative predictions regarding the relationships among perceptual variables (e.g., Eq. 1). These relationships spring from Bayes’ theorem: the product of a hypothesis’ likelihood and prior probability is proportional to its posterior probability. We liken the prior distribution to the observer’s expectation derived from experience, and the likelihood function to the sensation evoked by the stimulus (Figure 2). In our view, then, the Bayesian perceptual framework beautifully formalizes Helmholtz’s suggestion that “previous experiences act in conjunction with present sensations to produce a perceptual image” (Helmholtz, 1925).

Bayesian observers interpret sensory data in light of an internal model – a conception of the structure and statistics of the world. Bayesian perception is optimal when the observer’s internal model accurately represents the world – that is, when the observer’s prior distribution matches the stimulus distribution, and the observer’s likelihood function accurately reflects the process by which stimuli map to measurements (Figure 7). Unfortunately, the natural statistics of tactile stimuli have not been sufficiently characterized to constrain a prior distribution, nor is our knowledge of tactile sensorineural responses sufficient to specify the precise shape of a likelihood function. Accordingly, we fit a Gaussian prior and Gaussian likelihood to the human behavioral data. Subtle discrepancies between the human data and the model’s performance could result from our Gaussian assumptions. Future research is needed to determine the precise shapes of the priors and likelihoods used by individual participants. In any event, we speculate that a low-speed prior reflects the natural statistics of tactile stimuli, learned by humans through experience. If so, illusions such as the cutaneous rabbit may reveal the operation of an optimal observer who brings an expectation forged by real-world experience (the low-speed prior) into an artificial setting (the laboratory).

### The wide applicability of the low-speed-prior observer

Our Bayesian observer model may explain a variety of perceptual phenomena beyond the tactile illusions we have considered. One such phenomenon is the out-of-body rabbit illusion. In a clever experiment, Miyazaki et al. (2010) showed that humans perceived taps as hopping progressively along an aluminum bar resting across the index fingers of the hands, when in actuality the taps were delivered only to the points on the bar directly above each finger. To apply the model to this scenario, it is necessary only to know the observer’s likelihood function evoked by a tap to the bar: *p*(measurement | tap location along bar). An interesting twist here is that both hands might detect any single tap to the bar. This does not preclude the construction of a likelihood function; it simply requires consideration of the sensory input to both hands. For instance, a more intense vibration felt with the right hand would result in a likelihood function whose peak lies to the right of the bar’s center. Once the single tap likelihood functions are determined empirically, it would be straightforward to fit the model to the behavioral data with a low-speed prior. Of interest would be to compare the value of σ*_v_* so obtained to the value (∼10 cm/s) that fits the perception of trajectories delivered directly to the skin.

Our model provides insight into crossmodal interactions in length contraction illusions (Kawabe et al., 2008; Asai and Kanayama, 2012). In a 2-location, 3-tap rabbit paradigm, Asai and Kanayama (2012) demonstrated that the cutaneous rabbit was more consistently perceived when a visual flash occurred concurrently with, and at the typical illusory location of, the second tap. The model readily accommodates this cue-combination scenario. As shown in Figure 6, stochastic variability in the measurement causes trial-to-trial variability in the perceived location of either tap. Provided the Bayesian observer assumes that the concurrent visual and tactile measurements resulted independently from the same event, the observer’s likelihood function over that event’s location will be the product of the visual and tactile likelihoods. The visual measurement will therefore sharpen and shift the combined likelihood function toward the flash location, increasing the frequency with which the observer perceives the tactile stimulus to fall at that location. To test the model, one would first measure participants’ spatial uncertainty (σ_*s*_) in response to taps and flashes delivered in isolation. The model could then be used to make testable predictions regarding the perceptual influence of the flash.

Finally, our model may account for saltation illusions in both vision (Geldard, 1976; Lockhead et al., 1980; Khuu et al., 2011) and audition (Bremer et al., 1977; Shore et al., 1998; Getzmann, 2009). Provided the brain expects visual and auditory stimuli to move slowly, the model predicts pronounced length contraction when stimulus sequences traverse areas of poor spatial acuity (high σ_*s*_). In vision, this prediction has already been confirmed: the visual rabbit illusion occurs in response to peripheral but not central stimuli (Geldard, 1976). Furthermore, a low-speed prior has been implicated in visual motion perception (Weiss et al., 2002; Stocker and Simoncelli, 2006). Future experimental studies will assess the quantitative fit of our model to visual and auditory saltation illusions.

Despite its apparently wide applicability, we do not suggest that a low-speed prior alone can account for a majority of motion illusions. Interestingly, several visual motion phenomena (Nijhawan, 2002; Hubbard, 2005) involve endpoint overestimation similar to that caused by the low-acceleration prior that did not match the tactile data considered here (Figure 12B). Research is needed to clarify the conditions under which perception incorporates a low-acceleration prior.

### The percept as a combined pre- and post-dictive inference

Our Bayesian observer’s percept can be viewed as resulting from concomitant pre- and post-dictive inference. For instance, in two-tap trajectories, the first tap predicts the location of the second, while the second postdicts the location of the first (Figure 9). We suspect that Bayesian pre- and postdiction will be found to act together in many perceptual scenarios, whether or not these scenarios incorporate a low-speed prior. Indeed, it has already been reported that the two processes collaborate in the flash-lag effect (Rao et al., 2001; Soga et al., 2009), an illusion in which a brief visual flash placed alongside a moving object is perceived to lag behind the object.

By hypothesizing a link between spatial attention and σ_*s*_, as suggested by point localization experiments (Moore et al., 1999; O’Boyle et al., 2001), we have shown how attention can shape the relative influence of pre- and postdiction on the percept (Figure 9D). When attention is directed around the location of the first tap (σ_*s*1_ < σ_*s*2_), prediction dominates, and the second tap is perceived as asymmetrically displaced toward the first. When attention is directed around the location of the second tap (σ_*s*2_ < σ_*s*1_), postdiction dominates, and the first tap is perceived as asymmetrically displaced toward the second. Under conditions of imbalanced spatial attention, the trajectory midpoint is therefore perceived as shifted toward the attended location, as specified by Eq. (5). As the spatial attention balance is adjusted from one extreme to another, the model smoothly transitions between a percept influenced predominantly by prediction to one influenced predominantly by postdiction.

Researchers have often referred to the rabbit illusion as a post-dictive phenomenon, without mentioning the involvement of prediction (Bays et al., 2006; Blankenburg et al., 2006; van Wassenhove, 2009; Miyazaki et al., 2010; Asai and Kanayama, 2012). Indeed, initial work on the rabbit described only the perceptual displacement of the earlier tap(s) toward the later one(s) (Geldard and Sherrick, 1972), consistent with an exclusively postdictive process. However, it is clear from modern studies of the rabbit that both earlier and later taps undergo perceptual displacement – whether by equal distances or not (Kilgard and Merzenich, 1995; Flach and Haggard, 2006; Trojan et al., 2010). This supports our conclusion that the illusion involves concomitant predictive and postdictive inference.

Why did initial rabbit illusion investigations describe only the displacement of earlier taps toward later ones? In his three-tap “reduced rabbit” paradigm, Geldard (1982) stimulated with a “locator” (tap 1) followed at large ISI by an “attractee” (tap 2) at the same position, which he reported as perceptually displaced toward the subsequent “attractant” (tap 3) delivered at a different location. The participants’ report that tap 2 was perceptually displaced toward tap 3, but not vice versa, may have owed to the absence of a second locator tap placed at the position of tap 3. Without a locator tap for spatial comparison, participants may have been unaware that tap 3 was perceptually displaced. This hypothesis was considered and discarded by Geldard (1982) upon preliminary investigation, but Kilgard and Merzenich (1995), using a 4-tap paradigm that included a second locator tap, did find symmetric perceptual displacement of taps 2 and 3 (Figure 1C).

Alternatively, as demonstrated by Kilgard and Merzenich (1995) and modeled here, asymmetric rabbit percepts could reflect an imbalance in spatial attention (Figures 8 and 9D; Eq. 5). An interesting possibility is that – particularly during multi-tap sequences – participants have time to redistribute their spatial attention on the fly. When investigators randomize the direction of movement (up or down along the arm), the participants cannot know where to expect the first tap, so they presumably distribute their spatial attention equally. After the first tap has occurred, however, experienced participants will know where the trajectory is heading, and might direct their attention fully toward the upcoming final location. This would cause a decrease in σ_*s*_ at the final location, consequently shifting the percept toward that point (e.g., Figure 11D).

### Speculations regarding neural implementation

We have described two computational approaches by which our Bayesian observer could obtain its percept: either multi-dimensional inference (e.g., the two-dimensional inference shown in Figure 9A) or equivalent one-dimensional prediction-postdiction (Figure 9B). Which, if either, approach might the brain implement? The two approaches yield the same percept, but they scale very differently in difficulty as the number of taps increases. In the case of a sequence of *n* taps, the joint likelihood function, prior, and posterior would each require *n* dimensions. The neural representation of such multi-dimensional distributions would appear to pose considerable challenges. More plausibly, the brain could undertake one-dimensional predictive-postdictive inference recursively.

It is tempting to reinterpret the graphs in Figure 9 as plots of activity (e.g., spike rates) of a series of cortical neurons that represent the corresponding skin positions (*x*-axes). Under this interpretation, the predicted prior is a mound of cortical neural activity evoked by tap 1 that decays and broadens over time (Figure 9C). When the second tap initiates a second mound of cortical activity (the tap 2 likelihood function), the two mounds interact (e.g., through summation), resulting in a tap 2 percept that is shifted toward the tap 1 location. For trajectories with greater ISI, the tap 1 mound would have more time to decay, and would thus exert less influence over the tap 2 percept. This idea is similar to a model proposed by Flach and Haggard (2006). The idea is attractively simple; nevertheless, it seems able to account satisfactorily only for prediction, not postdiction. A more complex network model was proposed by Wiemer et al. (2000), but that model produces perceptual length dilation at large ISIs, a result contradicted by behavioral data.

Computationally, the perception of multi-tap sequences can be achieved with recursive predictive-postdictive Bayesian inference. The Kalman filter is an algorithm for recursive predictive inference (Haykin, 2001), for which plausible neural implementation schemes have been proposed (Deneve et al., 2007; Beck et al., 2011). Kalman smoothing combines the Kalman filter with recursive postdictive inference (Haykin, 2001). The percepts obtained by our Bayesian observer are identical to those that would result from an appropriately configured Kalman smoother (see “Multi-tap perception” in Appendix). Smoothing has already been implicated in the flash-lag effect (Rao et al., 2001) and proposed to contribute to a variety of motion illusions, including the rabbit (Grush, 2005), though to our knowledge a specific neural implementation for the Kalman smoother has not yet been proposed.

### Testable predictions

Our Bayesian observer model makes many testable predictions; we encourage other investigators to pursue these experimentally.

The model predicts that perceptual length contraction will be more pronounced on body areas with worse spatial acuity or – on a given body area – in response to stimuli that are harder to localize (e.g., weaker taps to the skin). Because σ_*s*_ can be independently manipulated and measured using single taps, the length contraction formula (Eq. 1) can be used to make specific testable predictions regarding the effect of body area or stimulus strength on the perception of two-tap trajectories.

Under conditions of imbalanced spatial attention, the model predicts that perceptual length contraction will occur in accordance with Eq. 4 and that the midpoint of the perceived two-tap trajectory will vary in accordance with Eq. 5. These predictions could be tested experimentally by independently measuring an observer’s σ_*s*1_ and σ_*s*2_ under different degrees of directed spatial attention, then measuring the trajectory percepts under the same conditions.

As explained above, the model can be used to make testable predictions regarding a variety of perceptual length contraction phenomena beyond those that we have modeled in this paper. These include the out-of-body rabbit, crossmodal influences on the rabbit percept, and the visual and auditory rabbit illusions.

We encourage readers to generate their own predictions by using our freely downloadable computer program, Leaping Lagomorphs (http://psych.mcmaster.ca/goldreich-lab/LL/Leaping_Lagomorphs.html). This convenient program implements the Bayesian observer, with either balanced or imbalanced spatial attention, and outputs its perception in response to any stimulus sequence that the user cares to enter.

## Conflict of Interest Statement

The authors declare that the research was conducted in the absence of any commercial or financial relationships that could be construed as a potential conflict of interest.

## Footnotes

^1^Neuroscientists may find it useful to conceive of the measurement as the location of the peak of evoked activity in the underlying receptor population (or its cortical equivalent), or more precisely as the maximal likelihood estimate of stimulus location, based on the neural response.

^2^We assume here that the observer veridically perceives the time between taps, such that temporal uncertainty is zero. Goldreich (2007) showed that temporal uncertainty exerts a negligible effect on the percept when stimuli occur on a skin region with poor spatial acuity, such as the forearm. Accordingly, here we confine ourselves to modeling stimuli on the forearm, which is also the skin region most often tested in experimental studies of the cutaneous tau and rabbit illusions.

^3^We note for reference that Goldreich (2007) defined the model’s free parameter as λ = σ_v_/σ_s_; thus, the lambda parameter in that paper is simply the reciprocal of the tau parameter.

^4^Note that “prior” in the Bayesian context does not imply “before” the stimulus occurs, but rather “independent of the measurement.” The predicted prior over tap 2’s position is constructed using all knowledge available to the observer except the tap 2 measurement, *x*_2*m*_. Similarly, the postdicted prior over tap 1’s position is constructed using all knowledge available to the observer except the tap 1 measurement, *x*_1*m*_.

^5^Although we have encountered a four-tap rabbit experiment (Figures 1C and 8), our approach was to consider the first and forth taps as mere reference points, so we modeled the perception of taps 2 and 3 only. Indeed, the first and forth taps in that sequence do not interact perceptually with the second and third, from which they are separated by large ISIs.

## Appendix

Here, we further develop mathematically, and offer new conceptual insights into, the *basic Bayesian observer* model put forth by Goldreich (2007). In the following seven sections, we: 1) specify the observer’s generative model, and derive the posterior probability density over tap trajectories and the perceptual length contraction formula; 2) generalize the derivation to include inhomogeneous spatial acuity caused by selective spatial attention; 3) consider useful one-dimensional reductions of the two-dimensional posterior density; 4) reformulate the observer’s percept as a combined predictive-postdictive inference; 5) model the perception of multi-tap sequences; 6) consider extensions of the model that incorporate additional sources of uncertainty; and 7) describe how we fit the model to human perceptual data.

### The Bayesian model

We consider here an observer whose goal is to perceive the locations of two-taps delivered to the skin in rapid succession. We assume that the observer has an internal generative model – a conception of the statistics of moving tactile stimuli – and that it interprets the stimulus sequence optimally within the context of its generative model. Briefly, the observer considers two taps that occur in rapid succession to result from a single moving object, and it considers that tactile objects tend to move slowly. Specifically, according to the generative model: (1) An object briefly touches the skin at a location, *x*_1_, drawn from a uniform density. (2) The object moves away from *x*_1_ with velocity *v*, drawn from a Gaussian density with mean zero and standard deviation σ_v_; at some elapsed time *t* (independent of *x*_1_), the object again briefly touches the skin, at location *x*_2_. (3) Noisy sensorineural activity evoked by each tap results in measured values for the tap positions, *x*_1*m*_ and *x*_2*m*_, drawn from Gaussian densities centered on the actual tap positions, *x*_1_ and *x*_2_, with standard deviations σ_s_.

#### Bayes’ formula

The observer’s goal is to infer the positions of the taps (*x*_1_, *x*_2_), which we refer to as the movement trajectory. We assume in this basic model that the observer perceives the time between taps, *t*, veridically. Thus, the observer knows *x*_1*m*_, *x*_2*m*_, and *t*, and wishes to infer *x*_1_ and *x*_2_. According to Bayes’ formula, the posterior over trajectories is proportional to the product of likelihood and prior:

(A1) $$p\left(x_{\text{1}}\text{,}\;x_{\text{2}}|x_{1m}\text{,}\;x_{2m}\text{,}\;t\right)\propto p\left(x_{1m}\text{,}\;x_{2m}|x_{\text{1}}\text{,}\;x_{\text{2}}\text{,}\;t\right)p\left(x_{1},x_{2}|t\right)$$

We now work out the observer’s prior and likelihood.

#### Prior probability density

The observer’s prior probability density over trajectories is:

(A2) $$p\left(x_{1},x_{2}|t\right)=p\left(x_{2}|x_{1},t\right)p\left(x_{1}|t\right)$$

Because *t* and *x*_1_ are independent, $p\left(x_{1}|t\right)=p\left(x_{1}\right)$, and this is a constant (*x*_1_ being drawn from a uniform distribution). Therefore, we can write more concisely:

(A3) $$p\left(x_{1},x_{2}|t\right)\propto p\left(x_{2}|x_{1},t\right)$$

We note that, given *x*_1_ and *t*, *x*_2_ is a function of the velocity, *v*:

(A4) $$x_{2}=x_{1}+vt$$

Thus, the probability that *v* resides in the infinitesimal region $\left(v\pm \frac{dv}{2}\right)$ is equal to the probability that *x*_2_ resides in the corresponding infinitesimal region $\left(x_{2}\pm \frac{dx_{2}}{2}\right)$:

(A5) $$p\left(x_{2}|\;x_{1},t\right)dx_{2}=p\left(v\right)dv$$

It follows that:

(A6) $$p\left(x_{2}|x_{1},\;t\right)=p\left(v\right)\left|\frac{dv}{dx_{2}}\right|=\frac{p\left(v\right)}{t}$$

Now recall that the observer has a low-velocity prior expectation:

(A7) $$p\left(v\right)=\frac{1}{\sqrt{2\pi }\sigma_{v}}\exp\left(-\frac{v^{2}}{2\sigma_{v}^{2}}\right)=\frac{1}{\sqrt{2\pi }\sigma_{v}}\exp\left(-\frac{{\left(\left(x_{2}-x_{1}\right)∕t\right)}^{2}}{2\sigma_{v}^{2}}\right)$$

Referring to Eqs A3, A6, and A7, we therefore have:

(A8) $$p\left(x_{1},x_{2}|t\right)\propto p\left(x_{2}|x_{1},t\right)=\frac{1}{\sqrt{2\pi }\sigma_{v}t}\exp\left(-\frac{{\left(x_{2}-x_{1}\right)}^{2}}{2{\left(\sigma_{v}t\right)}^{2}}\right)$$

The observer’s prior probability density over trajectories is proportional to a Gaussian distribution over the distance between taps, with mean zero and standard deviation $\sigma_{v}t$. Reflecting the low-speed prior, when the elapsed time, *t*, is large, a wide range of displacements is permissible; when *t* is shorter, the observer expects the two taps to more closely coincide spatially.

For future reference, we note that *x*_2_, like *x*_1_, is independent of *t*. We see this by integrating Eq. A8 with respect to *x*_1_:

(A9) $$p\left(x_{2}|t\right)=\underset{x_{1}}{\int }p\left(x_{1},x_{2}|t\right)dx_{1}\propto \underset{x_{1}}{\int }\frac{1}{\sqrt{2\pi }\sigma_{v}t}\exp\left(-\frac{{\left(x_{2}-x_{1}\right)}^{2}}{2{\left(\sigma_{v}t\right)}^{2}}\right)dx_{1}=1$$

Thus, *x*_2_ is independent of *t*, and, like *p*(*x*_1_), *p*(*x*_2_) is a constant. Eq. A8 shows that *x*_2_ is conditionally dependent on *t*, given *x*_1_.

#### Likelihood function

The tap positions measured by the observer, *x*_1*m*_ and *x*_2*m*_, are drawn independently from Gaussian densities centered on the actual tap positions, with standard deviations σ_*s*_. Therefore, the observer’s likelihood function is:

(A10) $$p\left(x_{1m},x_{2m}|x_{1},x_{2},t\right)=p\left(x_{1m}|x_{1}\right)p\left(x_{2m}|x_{2}\right)$$

where

(A11) $$p\left(x_{1m}|x_{1}\right)=\frac{1}{\sqrt{2\pi }\sigma_{s}}\exp\left(-\frac{{\left(x_{1m}-x_{1}\right)}^{2}}{2\sigma_{s}^{2}}\right)\;\;p\left(x_{2m}|x_{2}\right)=\frac{1}{\sqrt{2\pi }\sigma_{s}}\exp\left(-\frac{{\left(x_{2m}-x_{2}\right)}^{2}}{2\sigma_{s}^{2}}\right)$$

#### Posterior probability density

The observer uses Bayes’ formula (Eq. A1) to calculate the posterior density over trajectories. It is useful to express the posterior density in several ways. First, referring to Eqs A3 and A10, we see that Bayes’ formula can be rewritten:

(A12) $$p\left(x_{1},x_{2}|x_{1m},x_{2m},t\right)\propto p\left(x_{1m}|x_{1}\right)p\left(x_{2m}|x_{2}\right)p\left(x_{2}|x_{1},t\right)$$

Next, from Eqs A8 and A11, we have

(A13) $$p\left(x_{1},x_{2}|x_{1m},x_{2m},t\right)\propto \exp\left(-\left(\frac{{\left(x_{1m}-x_{1}\right)}^{2}+{\left(x_{2m}-x_{2}\right)}^{2}}{2\sigma_{s}^{2}}+\frac{{\left(x_{2}-x_{1}\right)}^{2}}{2{\left(\sigma_{v}t\right)}^{2}}\right)\right)$$

Finally, following some rearrangement, Eq. A13 can be written as a two-dimensional (2D) Gaussian distribution

(A14) $$p\left(x_{1},x_{2}|x_{1m},x_{2m},t\right)\propto \exp\left(-\frac{1}{2\left(1-\rho^{2}\right)}\left(\frac{{\left(x_{1}-x_{1^{*}}\right)}^{2}+{\left(x_{2}-x_{2^{*}}\right)}^{2}-2\rho \left(x_{1}-x_{1^{*}}\right)\left(x_{2}-x_{2^{*}}\right)}{\sigma^{2}}\right)\right)$$

where the posterior mode $\left(x_{1^{*}},x_{2^{*}}\right)$ is given by

$$\begin{array}{llll} x_{1^{*}}=x_{1m}\left(\frac{{\left(\sigma_{v}t\right)}^{2}+\sigma_{s}^{2}}{{\left(\sigma_{v}t\right)}^{2}+2\sigma_{s}^{2}}\right)+x_{2m}\left(\frac{\sigma_{s}^{2}}{{\left(\sigma_{v}t\right)}^{2}+2\sigma_{s}^{2}}\right) & & & \\ x_{2^{*}}=x_{1m}\left(\frac{\sigma_{s}^{2}}{{\left(\sigma_{v}t\right)}^{2}+2\sigma_{s}^{2}}\right)+x_{2m}\left(\frac{{\left(\sigma_{v}t\right)}^{2}+\sigma_{s}^{2}}{{\left(\sigma_{v}t\right)}^{2}+2\sigma_{s}^{2}}\right) & & & \end{array}$$

and the variance (σ^2^) and correlation coefficient (ρ) are given by:

$$\sigma^{2}=\sigma_{s}^{2}\frac{\sigma_{s}^{2}+{\left(\sigma_{v}t\right)}^{2}}{2\sigma_{s}^{2}+{\left(\sigma_{v}t\right)}^{2}}\;\rho =\frac{\sigma_{s}^{2}}{\sigma_{s}^{2}+{\left(\sigma_{v}t\right)}^{2}}$$

We assume that the observer reads out the posterior mode as the percept. Note that the perceived positions, $x_{1^{*}}\;\text{and}\;x_{2^{*}}$, are weighted averages of the measurements, *x*_1*m*_ and *x*_2*m*_. The perceived positions are drawn toward one another as the time between taps shortens, converging toward the measurement midpoint, (*x*_1*m*_ + *x*_2*m*_)/2, in the limit that *t* approaches zero. As *t* approaches infinity, by contrast, $x_{1^{*}}$ and $x_{2^{*}}$ approach the measured values, *x*_1*m*_ and *x*_2*m*_.

Subtracting $x_{1^{*}}$ from $x_{2^{*}}$, we find that the perceived distance between taps, $l^{*}=x_{2^{*}}-x_{1^{*}},$ relates to the measured distance, $l_{m}=x_{2m}-x_{1m}$, according to the formula:

(A15) $$l^{*}=x_{2^{*}}-x_{1^{*}}=\frac{x_{2m}-x_{1m}}{1+2{\left(\frac{\sigma_{s}}{\sigma_{v}t}\right)}^{2}}=\frac{l_{m}}{1+2{\left(\frac{\tau }{t}\right)}^{2}}$$

where we have defined the parameter tau as the ratio of the observer’s spatial uncertainty to the width of the low-speed prior: $\tau =\frac{\sigma_{s}}{\sigma_{v}}$.

Although the measured tap positions will vary stochastically from trial to trial, on average they will equal the actual tap positions. Thus, on average the perceived distance is related to the true distance, *l*, as:

(A16) $$l^{*}=\frac{l}{1+2{\left(\frac{\tau }{t}\right)}^{2}}$$

This is the perceptual length contraction formula, previously derived – using a different approach and expressed in a slightly different form – by Goldreich (2007).

### Generalization to inhomogeneous spatial uncertainty

So far we have assumed equal spatial uncertainty, σ_*s*_, at each point on the skin. Here, we consider the more general situation in which each tap may be associated with a different spatial uncertainty, σ_*s*1_ and σ_*s*2_, as might occur if the participant were to focus spatial attention on one skin region. In this case, the likelihood functions, Eq. A11, become:

(A17) $$p\left(x_{1m}|x_{1}\right)=\frac{1}{\sqrt{2\pi }\sigma_{s1}}\exp\left(-\frac{{\left(x_{1m}-x_{1}\right)}^{2}}{2\sigma_{s1}^{2}}\right)\;\;p\left(x_{2m}|x_{2}\right)=\frac{1}{\sqrt{2\pi }\sigma_{s2}}\exp\left(-\frac{{\left(x_{2m}-x_{2}\right)}^{2}}{2\sigma_{s2}^{2}}\right)$$

Consequently, the posterior density over tap positions (Eq. A13) becomes

(A18) $$p\left(x_{1},x_{2}|x_{1m},x_{2m},t\right)\propto \exp\left(-\left(\frac{{\left(x_{1m}-x_{1}\right)}^{2}}{2\sigma_{s1}^{2}}+\frac{{\left(x_{2m}-x_{2}\right)}^{2}}{2\sigma_{s2}^{2}}+\frac{{\left(x_{2}-x_{1}\right)}^{2}}{2{\left(\sigma_{v}t\right)}^{2}}\right)\right)$$

Following rearrangement, Eq. A18 can be re-written as a 2D Gaussian distribution,

(A19) $$p\left(x_{1},x_{2}|x_{1m},x_{2m},t\right)\propto \exp\left(-\frac{1}{2\left(1-\rho^{2}\right)}\left(\frac{{\left(x_{1}-x_{1^{*}}\right)}^{2}}{\sigma_{1}^{2}}+\frac{{\left(x_{2}-x_{2^{*}}\right)}^{2}}{\sigma_{2}^{2}}-\frac{2\rho \left(x_{1}-x_{1^{*}}\right)\left(x_{2}-x_{2^{*}}\right)}{\sigma_{1}\sigma_{2}}\right)\right)$$

where the posterior mode ($x_{1^{*}},x_{2^{*}}$) is given by

$$\begin{array}{llll} x_{1^{*}}=x_{1m}\left(\frac{{\left(\sigma_{v}t\right)}^{2}+\sigma_{s2}^{2}}{{\left(\sigma_{v}t\right)}^{2}+\sigma_{s1}^{2}+\sigma_{s2}^{2}}\right)+x_{2m}\left(\frac{\sigma_{s1}^{2}}{{\left(\sigma_{v}t\right)}^{2}+\sigma_{s1}^{2}+\sigma_{s2}^{2}}\right) & & & \\ x_{2^{*}}=x_{1m}\left(\frac{\sigma_{s2}^{2}}{{\left(\sigma_{v}t\right)}^{2}+\sigma_{s1}^{2}+\sigma_{s2}^{2}}\right)+x_{2m}\left(\frac{{\left(\sigma_{v}t\right)}^{2}+\sigma_{s1}^{2}}{{\left(\sigma_{v}t\right)}^{2}+\sigma_{s1}^{2}+\sigma_{s2}^{2}}\right) & & & \end{array}$$

and the variances $\left(\sigma_{1}^{2},\sigma_{2}^{2}\right)$ and correlation coefficient (ρ) are given by:

$$\sigma_{1}^{2}=\sigma_{s1}^{2}\frac{\sigma_{s2}^{2}+{\left(\sigma_{v}t\right)}^{2}}{\sigma_{s1}^{2}+\sigma_{s2}^{2}+{\left(\sigma_{v}t\right)}^{2}}\;\sigma_{2}^{2}=\sigma_{s2}^{2}\frac{\sigma_{s1}^{2}+{\left(\sigma_{v}t\right)}^{2}}{\sigma_{s1}^{2}+\sigma_{s2}^{2}+{\left(\sigma_{v}t\right)}^{2}}\;\rho =\frac{\sigma_{s1}\sigma_{s2}}{\sqrt{\left(\sigma_{s1}^{2}+{\left(\sigma_{v}t\right)}^{2}\right)\left(\sigma_{s2}^{2}+{\left(\sigma_{v}t\right)}^{2}\right)}}$$

It follows that

(A20) $$l^{*}=x_{2^{*}}-x_{1^{*}}=\frac{l_{m}}{1+\frac{\sigma_{s1}^{2}+\sigma_{s2}^{2}}{{\left(\sigma_{v}t\right)}^{2}}}=\frac{l_{m}}{1+2{\left(\frac{\sigma_{s\left(\text{rms}\right)}}{\sigma_{v}t}\right)}^{2}}$$

Thus, the uniform spatial uncertainty, σ_*s*_, of Eq. A15 is replaced by the root-mean-square of the uncertainty at the two locations:

$$\sigma_{s\left(\text{rms}\right)}=\sqrt{\frac{\sigma_{s1}^{2}+\sigma_{s2}^{2}}{2}}.$$

Interestingly, when $\sigma_{s1}\neq \sigma_{s2},$ the midpoint of the perceived trajectory no longer coincides with the midpoint of the measured trajectory. From the expressions (Eq. A19) for $x_{1^{*}}$ and $x_{2^{*}}$ it is easily shown that the shift, $\Delta_{\text{midpt}},$ in the perceived trajectory midpoint away from the measured trajectory midpoint is:

(A21) $$\Delta_{\text{midpt}}=\frac{x_{1^{*}}+x_{2^{*}}}{2}-\frac{x_{1m}+x_{2m}}{2}=\frac{l_{m}}{2}\left(\frac{\sigma_{s1}^{2}-\sigma_{s2}^{2}}{{\left(\sigma_{v}t\right)}^{2}+\sigma_{s1}^{2}+\sigma_{s2}^{2}}\right)$$

### One-dimensional reductions

The two-dimensional joint (*x*_1_, *x*_2_) posterior density (Eq. A19) fully represents the observer’s belief distribution over stimulus trajectories, and it captures dependencies between the variables. Nevertheless, it can be useful to express the observer’s belief about a single parameter of interest, although this entails a loss of information about dependencies. One such parameter of interest is the length, *l*, between taps. Other parameters of interest are the tap positions, *x*_1_ and *x*_2_, considered individually. Here we derive the observer’s one-dimensional posterior densities over each of these parameters.

#### Posterior density over trajectory length

The posterior over trajectory length, *l* = *x*_2_ − *x*_1_, can be found by integrating across the joint posterior:

(A22) $$p\left(l|x_{1m},x_{2m},t\right)=\underset{x_{1}}{\int }p\left(x_{1},x_{2}=l+x_{1}|x_{1m},x_{2m},t\right)dx_{1}$$

The posterior over *l* can also be found by noting that, from Eq. A8, the observer’s prior over *l* is:

(A23) $$p\left(l|t\right)=\frac{1}{\sqrt{2\pi }\sigma_{v}t}\exp\left(-\frac{l^{2}}{2{\left(\sigma_{v}t\right)}^{2}}\right)$$

Further, from Eq. A17, we see that the observer’s displacement measurement*, l_m_* = *x*_2*m*_ − *x*_1*m*_, is normally distributed with mean *l* and variance $\sigma_{s1}^{2}+\sigma_{s2}^{2}:$

(A24) $$p\left(l_{m}|l\right)=\frac{1}{\sqrt{2\pi \left(\sigma_{s1}^{2}+\sigma_{s2}^{2}\right)}}\exp\left(-\frac{{\left(l_{m}-l\right)}^{2}}{2\left(\sigma_{s1}^{2}+\sigma_{s2}^{2}\right)}\right)$$

Thus, by Bayes’ rule, the posterior over *l* is proportional to the product of these two Gaussian densities:

(A25) $$p\left(l|l_{m},t\right)\propto p\left(l_{m}|l,t\right)p\left(l|t\right)$$

The result is a Gaussian posterior density with mean and variance given by:

(A26) $$\mu_{l\;\text{posterior}}=\frac{l_{m}}{1+\frac{\sigma_{s1}^{2}+\sigma_{s2}^{2}}{{\left(\sigma_{v}t\right)}^{2}}},\;\sigma_{l\;\text{posterior}}^{2}=\frac{1}{\frac{1}{\sigma_{s1}^{2}+\sigma_{s2}^{2}}+\frac{1}{{\left(\sigma_{v}t\right)}^{2}}}$$

The mean of the posterior over *l* is again the length contraction formula, Eq. A20. The variance of the posterior over *l* is smaller than the variance of *l_m_*, given *l*. For this reason, the observer’s length percept is more accurate than the length measurement (see Figure 7).

#### Marginal posterior densities over *x*_1_ and *x*_2_

To express the observer’s belief about each tap’s position individually, we can integrate the joint posterior along *x*_2_ to find the marginal posterior over *x*_1_, and integrate the joint posterior along *x*_1_ to find the marginal posterior over *x*_2_:

(A27) $$\begin{array}{c} p\left(x_{1}|x_{1m},x_{2m},t\right)=\underset{x_{2}}{\int }p\left(x_{1},x_{2}|x_{1m},x_{2m},t\right)dx_{2}\\ p\left(x_{2}|x_{1m},x_{2m},t\right)=\underset{x_{1}}{\int }p\left(x_{1},x_{2}|x_{1m},x_{2m},t\right)dx_{1} \end{array}$$

Because the joint posterior density is a 2D Gaussian (Eq. A19), the marginalization integrals (Eq. A27) have simple solutions:

(A28) $$\begin{array}{c} p\left(x_{1}|x_{1m},x_{2m},t\right)=\frac{1}{\sqrt{2\pi }\sigma_{1}}\exp\left(-\frac{{\left(x_{1}-x_{1^{*}}\right)}^{2}}{2\sigma_{1}^{2}}\right)\\ p\left(x_{2}|x_{1m},x_{2m},t\right)=\frac{1}{\sqrt{2\pi }\sigma_{2}}\exp\left(-\frac{{\left(x_{2}-x_{2^{*}}\right)}^{2}}{2\sigma_{2}^{2}}\right) \end{array}$$

### The prediction-postdiction formulation

Here, we show that the observer’s marginal posterior over *x*_2_ can be equivalently derived from predictive inference: upon observing tap 1, the observer predicts (infers forward in time) a prior over tap 2; the observer then combines this *predicted prior* with the tap 2 likelihood to obtain the posterior over *x*_2_. Conversely, the marginal posterior over *x*_1_ can be derived from postdictive inference: upon observing tap 2, the observer postdicts (infers backward in time) a prior over tap 1; the observer then combines this *postdicted prior* with the tap 1 likelihood to obtain the posterior over *x*_1_.

#### Predicting tap 2 upon observing tap 1

Replacing the integrand in lower Eq. A27 with the expression from Eq. A1, we have:

(A29) $$p\left(x_{2}|x_{1m},x_{2m},t\right)\propto \underset{x_{1}}{\int }p\left(x_{1m},x_{2m}|x_{1},x_{2},t\right)p\left(x_{1},x_{2}|t\right)dx_{1}$$

Further expanding the integrand, we have:

(A30) $$p\left(x_{2}|x_{1m},x_{2m},t\right)\propto \underset{x_{1}}{\int }p\left(x_{1m}|x_{1}\right)p\left(x_{2m}|x_{2}\right)p\left(x_{2}|x_{1},t\right)p\left(x_{1}\right)dx_{1}$$

Because $p\left(x_{2m}|x_{2}\right)$ does not depend on *x*_1_, we move it outside the integral. Thus, we have:

(A31) $$p\left(x_{2}|x_{1m},x_{2m},t\right)\propto p\left(x_{2m}|x_{2}\right)\underset{x_{1}}{\int }p\left(x_{1m}|x_{1}\right)p\left(x_{2}|x_{1},t\right)p\left(x_{1}\right)dx_{1}$$

Now we note that, according to Bayes’ formula:

(A32) $$p\left(x_{1m}|x_{1}\right)p\left(x_{1}\right)\propto p\left(x_{1}|x_{1m}\right)$$

Substituting Eq. A32 into Eq. A31 yields:

(A33) $$p\left(x_{2}|x_{1m},x_{2m},t\right)\propto p\left(x_{2m}|x_{2}\right)\underset{x_{1}}{\int }p\left(x_{2}|x_{1},t\right)p\left(x_{1}|x_{1m}\right)dx_{1}$$

Equation A33 is Bayes’ formula for the tap 2 position, *x*_2_. It states that the marginal posterior density over *x*_2_ is proportional to the product of the tap 2 likelihood, $p\left(x_{2m}|x_{2}\right)$, and the tap 2 *predicted prior density*,

(A34) $$p\left(x_{2}|x_{1m},t\right)=\underset{x_{1}}{\int }p\left(x_{2}|x_{1},t\right)p\left(x_{1}|x_{1m}\right)dx_{1}$$

The predicted prior projects belief forwards in time. It reflects the observer’s beliefs about tap 2, given the tap 1 measurement and the elapsed time. Based on *x*_1*m*_, the observer can generate a posterior over tap 1, *p*(*x*_1_|*x*_1*m*_). The predicted prior over a particular tap 2 position is then calculated by integrating across every possible tap 1 the product of this tap 1 posterior with the probability that the particular tap 2 will follow.

#### Postdicting tap 1 upon observing tap 2

Replacing the integrand in upper Eq. A27 with the expression from Eq. A1, we have:

(A35) $$p\left(x_{1}|x_{1m},x_{2m},t\right)\propto \underset{x_{2}}{\int }p\left(x_{1m},x_{2m}|x_{1},x_{2},t\right)p\left(x_{1},x_{2}|t\right)dx_{2}$$

Further expanding the integrand, we have:

(A36) $$p\left(x_{1}|x_{1m},x_{2m},t\right)\propto \underset{x_{2}}{\int }p\left(x_{1m}|x_{1}\right)p\left(x_{2m}|x_{2}\right)p\left(x_{1}|x_{2},t\right)p\left(x_{2}\right)dx_{2}$$

Because *p*(*x*_1*m*_|*x*_1_) does not depend on *x*_2_, we move it outside the integral. Thus, we have:

(A37) $$p\left(x_{1}|x_{1m},x_{2m},t\right)\propto p\left(x_{1m}|x_{1}\right)\underset{x_{2}}{\int }p\left(x_{2m}|x_{2}\right)p\left(x_{1}|x_{2},t\right)p\left(x_{2}\right)dx_{2}$$

Now we note that, according to Bayes’ formula:

(A38) $$p\left(x_{2m}|x_{2}\right)p\left(x_{2}\right)\propto p\left(x_{2}|x_{2m}\right)$$

Substituting Eq. A38 into Eq. A37 yields:

(A39) $$p\left(x_{1}|x_{1m},x_{2m},t\right)\propto p\left(x_{1m}|x_{1}\right)\underset{x_{2}}{\int }p\left(x_{1}|x_{2},t\right)p\left(x_{2}|x_{2m}\right)dx_{2}$$

Equation A39 is Bayes’ formula for the tap 1 position, *x*_1_. It states that the marginal posterior density over *x*_1_ is proportional to the product of the tap 1 likelihood, *p*(*x*_1*m*_|*x*_1_), and the tap 1 *postdicted prior density*,

(A40) $$p\left(x_{1}|x_{2m},t\right)=\underset{x_{2}}{\int }p\left(x_{1}|x_{2},t\right)p\left(x_{2}|x_{2m}\right)dx_{2}$$

The postdicted prior projects belief backwards in time. It reflects the observer’s beliefs about tap 1, given the tap 2 measurement and the elapsed time. Based on *x*_2*m*_, the observer can generate a posterior over tap 2, *p*(*x*_2_|*x*_2*m*_). The postdicted prior over a particular tap 1 position is then calculated by integrating across every possible tap 2 the product of this tap 2 posterior with the probability that the particular tap 1 preceded.

#### Formulas for the predicted and postdicted prior densities

We now solve the predicted and postdicted prior integrals (Eqs A34 and A40). To find the predicted prior, we substitute from Eqs A8 and A17 left, into Eq. A34:

(A41) $$\begin{array}{ll} p\left(x_{2}|x_{1m},t\right) & =\underset{x_{1}}{\int }\frac{1}{\sqrt{2\pi }\sigma_{v}t}\exp\left(-\frac{{\left(x_{2}-x_{1}\right)}^{2}}{2{\left(\sigma_{v}t\right)}^{2}}\right)\frac{1}{\sqrt{2\pi }\sigma_{s1}}\exp\left(-\frac{{\left(x_{1m}-x_{1}\right)}^{2}}{2\sigma_{s1}^{2}}\right)dx_{1}\\ & =\frac{1}{2\pi \sigma_{v}t\sigma_{s1}}\underset{x_{1}}{\int }\exp\left[-\left(\frac{{\left(x_{2}-x_{1}\right)}^{2}}{2{\left(\sigma_{v}t\right)}^{2}}+\frac{{\left(x_{1m}-x_{1}\right)}^{2}}{2\sigma_{s1}^{2}}\right)\right]dx_{1} \end{array}$$

We note that, upon much rearrangement:

(A42) $$\frac{{\left(x_{2}-x_{1}\right)}^{2}}{2{\left(\sigma_{v}t\right)}^{2}}+\frac{{\left(x_{1m}-x_{1}\right)}^{2}}{2\sigma_{s1}^{2}}=\frac{{\left(\sigma_{v}t\right)}^{2}+\sigma_{s1}^{2}}{2\sigma_{s1}^{2}{\left(\sigma_{v}t\right)}^{2}}{\left(x_{1}-\frac{x_{2}\sigma_{s1}^{2}+x_{1m}{\left(\sigma_{v}t\right)}^{2}}{{\left(\sigma_{v}t\right)}^{2}+\sigma_{s1}^{2}}\right)}^{2}+\frac{1}{2}\left(\frac{{\left(x_{2}-x_{1m}\right)}^{2}}{{\left(\sigma_{v}t\right)}^{2}+\sigma_{s1}^{2}}\right)$$

Thus, Eq. A41 becomes,

(A43) $$p\left(x_{2}|x_{1m},t\right)=\frac{1}{2\pi \sigma_{v}t\sigma_{s1}}\exp\left(-\frac{{\left(x_{2}-x_{1m}\right)}^{2}}{2\left({\left(\sigma_{v}t\right)}^{2}+\sigma_{s1}^{2}\right)}\right)\underset{x_{1}}{\int }\exp\left(-\frac{{\left(x_{1}-\frac{x_{2}\sigma_{s1}^{2}+x_{1m}{\left(\sigma_{v}t\right)}^{2}}{{\left(\sigma_{v}t\right)}^{2}+\sigma_{s1}^{2}}\right)}^{2}}{\frac{2\sigma_{s1}^{2}{\left(\sigma_{v}t\right)}^{2}}{{\left(\sigma_{v}t\right)}^{2}+\sigma_{s1}^{2}}}\right)dx_{1}$$

The integrand is a Gaussian function with standard deviation

$$\frac{\sigma_{s1}\sigma_{v}t}{\sqrt{{\left(\sigma_{v}t\right)}^{2}+\sigma_{s1}^{2}}}.$$

Because the integral of an un-normalized Gaussian function of standard deviation σ is $\sqrt{2\pi }\sigma$, Eq. A43 simplifies to:

(A44) $$p\left(x_{2}|x_{1m},t\right)=\frac{1}{2\pi \sigma_{v}t\sigma_{s1}}\exp\left(-\frac{{\left(x_{2}-x_{1m}\right)}^{2}}{2\left({\left(\sigma_{v}t\right)}^{2}+\sigma_{s1}^{2}\right)}\right)\frac{\sqrt{2\pi }\sigma_{s1}\sigma_{v}t}{\sqrt{{\left(\sigma_{v}t\right)}^{2}+\sigma_{s1}^{2}}}$$

Therefore, the predicted prior density over *x*_2_ is

(A45) $$p\left(x_{2}|x_{1m},t\right)=\frac{1}{\sqrt{2\pi \left({\left(\sigma_{v}t\right)}^{2}+\sigma_{s1}^{2}\right)}}\exp\left(-\frac{{\left(x_{2}-x_{1m}\right)}^{2}}{2\left({\left(\sigma_{v}t\right)}^{2}+\sigma_{s1}^{2}\right)}\right)$$

That is, the predicted prior is a Gaussian with mean and variance

(A46) $$\mu_{\text{pre}}=x_{1m}\;\sigma_{\text{pre}}^{2}={\left(\sigma_{v}t\right)}^{2}+\sigma_{s1}^{2}$$

A similar derivation reveals that the postdicted prior density over *x*_1_ is

(A47) $$p\left(x_{1}|x_{2m},t\right)=\frac{1}{\sqrt{2\pi \left({\left(\sigma_{v}t\right)}^{2}+\sigma_{s2}^{2}\right)}}\exp\left(-\frac{{\left(x_{1}-x_{2m}\right)}^{2}}{2\left({\left(\sigma_{v}t\right)}^{2}+\sigma_{s2}^{2}\right)}\right)$$

That is, the postdicted prior is a Gaussian with mean and variance

(A48) $$\mu_{\text{post}}=x_{2m}\;\sigma_{\text{post}}^{2}={\left(\sigma_{v}t\right)}^{2}+\sigma_{s2}^{2}$$

### Multi-tap perception

So far, we have considered trajectories composed of just two taps. An interesting question arises in modeling the perception of multi-tap stimuli: is the observer’s generative model (a) a direct extension of the one we have considered here, such that a zero-mean low-speed prior applies independently to each pair of consecutive taps, or (b) does the observer expect velocity to be consistent across the multi-tap trajectory, such that the prior applied to each tap pair might be a Gaussian centered on the velocity of the preceding pair (a zero-mean low-acceleration prior)?

Considering trajectories with an arbitrary number of taps, *n*, and permitting inhomogeneous spatial acuity, possibilities (a) and (b) result in the following generalizations of Eq. A18:

(a) (A49) $$p\left(\left\{x_{i}\right\}|\left\{x_{im}\right\},\left\{t_{i}\right\}\right)\propto \exp\left(-\left(\overset{n}{\underset{i=1}{\sum }}\frac{{\left(x_{im}-x_{i}\right)}^{2}}{2\sigma_{si}^{2}}+\overset{n-1}{\underset{i=1}{\sum }}\frac{{\left(x_{i+1}-x_{i}\right)}^{2}}{2{\left(\sigma_{v}t_{i}\right)}^{2}}\right)\right)$$
(b) (A50) $$p\left(\left\{x_{i}\right\}|\left\{x_{im}\right\},\left\{t_{i}\right\}\right)\propto \exp\left(-\left(\overset{n}{\underset{i=1}{\sum }}\frac{{\left(x_{im}-x_{i}\right)}^{2}}{2\sigma_{si}^{2}}+\frac{{\left(x_{2}-x_{1}\right)}^{2}}{2{\left(\sigma_{v}t_{1}\right)}^{2}}+\overset{n-1}{\underset{i=2}{\sum }}\frac{{\left(\frac{x_{i+1}-x_{i}}{t_{i}}-\frac{x_{i}-x_{i-1}}{t_{i-1}}\right)}^{2}}{2\sigma_{v}^{2}}\right)\right)$$

Here {*x_i_*} refers to the set of tap positions, *x*_1_, *x*_2_, … *x_n_*; {*x_im_*} to the corresponding set of measurements; {*t_i_*} to the set of times elapsed between each tap *i* and tap *i* + 1; and σ*_si_* to the spatial uncertainty associated with tap *i*.

The observer’s percept $\left\{x_{i}^{*}\right\}$ in case (a) or (b) can be found by taking partial derivatives of Eq. A49 or Eq. A50 with respect to each of the {*x_i_*}, setting these to zero, and solving the simultaneous equations. We used this method to find the percepts depicted in Figures 10 and 11 [case (a)] and Figure 12 [case (b)].

Alternatively, the identical percept can be found through Kalman smoothing (Haykin, 2001), a recursive extension of the predictive-postdictive formulation described above. The Kalman smoother consists of an iterative forward (predictive) pass through the stimulus sequence, followed by a backward (postdictive) pass. For model (a), the algorithm for the forward pass (the Kalman *filter*) is:

(A51) $$\begin{array}{ll} K_{i} & =\frac{\sigma_{i-1|i-1}^{2}+{\left(\sigma_{v}t\right)}^{2}}{\sigma_{i-1|i-1}^{2}+{\left(\sigma_{v}t\right)}^{2}+\sigma_{s}^{2}}\\ {\hat{x}}_{i|i} & ={\hat{x}}_{i-1|i-1}+K_{i}\left(x_{im}-{\hat{x}}_{i-1|i-1}\right)\\ \sigma_{i|i}^{2} & =\left(1-K_{i}\right)\left(\sigma_{i-1|i-1}^{2}+{\left(\sigma_{v}t\right)}^{2}\right) \end{array}$$

Here, *K_i_* is the *Kalman gain* at time *i*; the notation ${\hat{x}}_{i|j}$ refers to the estimated position of tap *i* based on all taps up to and including tap *j*; and $\sigma_{i|j}^{2}$ is the variance of that estimate. The filter is initialized at the first tap, with ${\hat{x}}_{1|1}=x_{1m},\;\sigma_{1|1}^{2}=\sigma_{s}^{2}$, and runs forward until tap *n* is reached. The Rauch-Tung-Striebel algorithm for the subsequent backward pass is:

(A52) $$\begin{array}{ll} C_{i} & =\frac{\sigma_{i|i}^{2}}{\sigma_{i|i}^{2}+{\left(\sigma_{v}t\right)}^{2}}\\ {\hat{x}}_{i|n} & ={\hat{x}}_{i|i}+C_{i}\left({\hat{x}}_{i+1|n}-{\hat{x}}_{i|i}\right)\\ \sigma_{i|n}^{2} & =\sigma_{i|i}^{2}+C_{i}^{2}\left(\sigma_{i+1|n}^{2}-\sigma_{i|i}^{2}-{\left(\sigma_{v}t\right)}^{2}\right) \end{array}$$

We verified that Eqs A51 and A52 yielded the same percepts plotted in Figures 10 and 11.

### Extensions

Although skin is a two-dimensional surface, we have so far considered only a single position axis, *x*, along which stimuli occur. In essence, we have assumed that the orthogonal, *y* coordinate, of the taps is a known constant. We have also assumed that the time, *t*, is known. Each of these restrictions can be removed.

#### Two-dimensional movement

A more realistic generative model would allow stimuli to move in any direction along a two-dimensional skin surface. To accomplish this, we can adopt an (*x*,*y*) Cartesian coordinate system in which the orthogonal components of the velocity vector are independently specified by low-speed priors:

(A53) $$\begin{array}{c} p\left(v_{x}\right)=\frac{1}{\sqrt{2\pi }\sigma_{v}}\exp\left(-\frac{v_{x}^{2}}{2\sigma_{v}^{2}}\right)=\frac{1}{\sqrt{2\pi }\sigma_{v}}\exp\left(-\frac{{\left(\left(x_{2}-x_{1}\right)/t\right)}^{2}}{2\sigma_{v}^{2}}\right)\\ p\left(v_{y}\right)=\frac{1}{\sqrt{2\pi }\sigma_{v}}\exp\left(-\frac{v_{y}^{2}}{2\sigma_{v}^{2}}\right)=\frac{1}{\sqrt{2\pi }\sigma_{v}}\exp\left(-\frac{{\left(\left(y_{2}-y_{1}\right)/t\right)}^{2}}{2\sigma_{v}^{2}}\right) \end{array}$$

The tap 1 and 2 likelihood functions generalize to:

(A54) $$\begin{array}{c} p\left(x_{1m},y_{1m}|x_{1},y_{1}\right)=\frac{1}{\sqrt{2\pi }\sigma_{s1}}\exp\left(-\frac{{\left(x_{1m}-x_{1}\right)}^{2}+{\left(y_{1m}-y_{1}\right)}^{2}}{2\sigma_{s1}^{2}}\right)\\ p\left(x_{2m},y_{2m}|x_{2},y_{2}\right)=\frac{1}{\sqrt{2\pi }\sigma_{s2}}\exp\left(-\frac{{\left(x_{2m}-x_{2}\right)}^{2}+{\left(y_{2m}-y_{2}\right)}^{2}}{2\sigma_{s2}^{2}}\right) \end{array}$$

The posterior over trajectories then takes the form:

(A55) $$\begin{array}{llll} & p\left(x_{1},y_{1},x_{2},y_{2}|x_{1m},y_{1m},x_{2m},y_{2m},t\right) & & \\ & \;\propto \exp\left(-\left(\frac{{\left(x_{1m}-x_{1}\right)}^{2}+{\left(y_{1m}-y_{1}\right)}^{2}}{2\sigma_{s1}^{2}}+\frac{{\left(x_{2m}-x_{2}\right)}^{2}+{\left(y_{2m}-y_{2}\right)}^{2}}{2\sigma_{s2}^{2}}+\frac{{\left({\left(x_{2}-x_{1}\right)}^{2}+{\left(y_{2}-y_{1}\right)}^{2}\right)}^{2}}{2{\left(\sigma_{v}t\right)}^{2}}\right)\right) & \end{array}$$

It is straightforward to show that the length contraction formula resulting from Eq. A55 is identical to Eq. A20. Indeed, if we define the *x*-axis as the axis along which the tap measurements lie, then marginalization of Eq. A55 over *y*_1_ and *y*_2_ recovers the posterior density Eq. A18.

#### Temporal uncertainty

Our model has assumed that the time between stimuli, *t*, is perceived veridically. This assumption can be removed. Goldreich (2007) showed that the Bayesian observer with temporal uncertainty tends to overestimate *t* in addition to underestimating *l*. Thus, the Bayesian observer can model time dilation as well as length contraction illusions.

### Fitting to human perceptual data

We found the value of tau that minimized the mean-squared error (MSE) between human and model performance. This was done separately for the perceptual data from Marks et al. (1982), Lechelt and Borchert (1977), and Kilgard and Merzenich (1995), shown in Figures 1A–C, and for the data from Helson and King (1931), shown in Figure 10.

The data of Helson and King (1931) required some processing prior to the fitting procedure. We fit the data reported in Tables 2–6 of Helson and King (1931). In those experiments, on each trial the participant reported whether the second spatial interval was perceived to be shorter than, equal to, or longer than the first interval (which was fixed at 3 cm). To fit these data, we first transformed them into an equivalent two-alternative forced-choice format by distributing each participant’s “equal” responses evenly to the “shorter” and “longer” response categories. We then fit each participant’s transformed data (proportion “*l*_2_ is longer” responses) at each *t*_2_ setting with a Weibull psychometric function:

$$\Psi_{a,b,\gamma ,\delta }\left(l_{2}\right)=\left(1-\delta \right)\left[\gamma +\left(1-\gamma \right)\left(1-2^{-{\left(\frac{l_{2}-3cm}{a}\right)}^{b}}\right)\right]+\frac{\delta }{2}$$

Here δ is a lapse rate, γ is the probability that the concentrating participant would answer “*l*_2_ is longer” when in fact *l*_2_ = *l*_1_ (i.e., 3 cm), *a* is a position parameter, and *b* is a slope parameter. We found the maximum likelihood parameter settings, and from them read off the point of subjective equality (PSE: *l*_2_ that the participant judged longer than *l*_1_ with 50% probability). We fit the Bayesian observer’s tau to minimize the MSE between its performance and the average PSE of the six human participants across the five *t*_2_ values tested by Helson and King (1931). Before doing these fits, we discarded the data from one of the six participants on one of the five *t*_2_ points: “Observer B” of Helson and King (1931) did not have a valid PSE at *t*_2_ = 0.25 s because that participant’s transformed “*l*_2_ is longer” response proportion was greater than 50% at all *l*_2_ values.
